# Post–operative glioblastoma cancer cell distribution in the peritumoural oedema

**DOI:** 10.3389/fonc.2024.1447010

**Published:** 2024-12-12

**Authors:** Andrei Ciprian Macarie, Szabolcs Suveges, Mohamed Okasha, Kismet Hossain-Ibrahim, J. Douglas Steele, Dumitru Trucu

**Affiliations:** ^1^ Division of Mathematics, University of Dundee, Dundee, United Kingdom; ^2^ Division of Neuroscience, School of Medicine, University of Dundee, Dundee, United Kingdom; ^3^ Department of Neurosurgery, Ninewells Hospital and School of Medicine, National Health Service (NHS) Tayside, Dundee, United Kingdom

**Keywords:** multiscale modelling, cancer invasion, glioblastoma, chemotherapy, radiotherapy, surgery, 3D computational modelling, MRI scans

## Abstract

Glioblastoma multiforme (GBM), the most aggressive primary brain tumour, exhibits low survival rates due to its rapid growth, infiltrates surrounding brain tissue, and is highly resistant to treatment. One major challenge is oedema infiltration, a fluid build-up that provides a path for cancer cells to invade other areas. MRI resolution is insufficient to detect these infiltrating cells, leading to relapses despite chemotherapy and radiotherapy. In this work, we propose a new multiscale mathematical modelling method, to explore the oedema infiltration and predict tumour relapses. To address tumour relapses, we investigated several possible scenarios for the distribution of remaining GBM cells within the oedema after surgery. Furthermore, in this computational modelling investigation on tumour relapse scenarios were investigated assuming the presence of clinically relevant chemo-radio therapy, numerical results suggest that a higher concentration of GBM cells near the surgical cavity edge led to limited spread and slower progression of tumour relapse. Finally, we explore mathematical and computational avenues for reconstructing relevant shapes for the initial distributions of GBM cells within the oedema from available MRI scans. The results obtained show good overlap between our simulation and the patient’s serial MRI scans taken 881 days into the treatment. While still under analytical investigation, this work paves the way for robust reconstruction of tumour relapses from available clinical data.

## 1 Introduction

Glioblastoma multiforme (GBM) is a devastating and highly invasive brain tumour that presents a significant treatment challenge. Despite the best efforts of medical professionals, the 5–year survival rate for patients with GBM is only 7.2% (1, 2). To improve treatment outcomes, researchers have been exploring new approaches to tackling this aggressive disease. One promising avenue of investigation is the use of mathematical models to simulate tumour evolution and explore potential new treatment strategies (3–7).

GBM is typically treated with surgical resection if possible, followed by chemoradiotherapy. The Stupp protocol is the standard of care treatment regimen that involves a total of 60 grays (abbreviated as *Gy*, and representing the unit of measurement for absorbed radiation) of radiotherapy delivered in daily doses of 2 Gy over 6 weeks, along with the chemotherapy drug Temozolomide (TMZ). During radiotherapy, patients take 75 mg of TMZ per square meter of body surface area every day for 7 days a week. After radiotherapy is completed, TMZ (adjuvant) is given in 6 cycles of 150–200 mg per square meter for 5 days every 28 days (8).

Treating GBM is a formidable challenge due to several factors. Even after maximal surgical resection and adherence to the Stupp protocol, approximately 90% of patients experience local recurrences (9–12). Another significant challenge is the high infiltration and heterogeneity of GBM, which makes it difficult to identify tumour margins accurately. GBM grows with microscopic finger–like projections that extend beyond what MRI scans (the gold standard for brain tumour imaging) can detect (2). Furthermore, GBM cells invade the brain through the peritumoural oedema (PTE), a condition in which fluid accumulates in the extracellular spaces of brain tissue surrounding the tumour. PTE is formed by tumour cells, reactive astrocytes, and inflammatory cells. The infiltrating GBM cells in the PTE are phenotypically distinct from those isolated from the corresponding mass. Residual GBM cells located at the resection margin are known to proliferate more quickly and be more invasive than GBM cells found in the tumour center (9, 13). Therefore, it is crucial to examine the PTE as this could lead to tumour recurrences (14).

The limited effectiveness of traditional GBM treatments underscores the need for innovative approaches (4, 15). In recent years, mathematical models have emerged as a promising tool for gaining insights into GBM tumour growth and progression (7, 16–21). By incorporating clinical data and biological parameters, mathematical models can provide a more comprehensive understanding of tumour behaviour than traditional experimental techniques alone (22, 23). However, most of these studies, whether 2D or 3D, are limited to simulating tumour growth on one spatio–temporal scale, *i.e.*, focused on modelling tumour growth based mostly on the macro–scale dynamics (24–28). Nevertheless, significant progress has been made in developing multiscale moving boundary modelling and computational frameworks for tumour growth (3, 6, 7, 29–31). As detailed below, the combination of these modelling approaches paves the way for the work discussed here.

In this work we aim to explore the distribution of GBM cells within the oedema. The underlying motivation for this is the understanding of the relationship between the spatial distribution of cancer cells within oedema that remain post–surgery and the likelihood of post–surgical tumour recurrence. This will combine novel mathematical multiscale moving boundary modelling with NHS clinical data assimilation using MRI scans from a single patient with diagnosed GBM. We explore two scenarios: the first utilizes a standard mollifier to describe cell distribution inside the oedema, while the second uses a Gaussian distribution.

This paper presents a multiscale moving boundary model for simulating GBM evolution, incorporating treatment effects and clinical data. After introducing our multiscale modelling for GBM progression, we formulate our tumour relapse hypothesis and outline the mathematical and computational strategy for clinical data inversion (*i.e.*, assimilate MRI images within our modelling to enable tumour recurrence predictions). Details about prospectively collected MRI scans (from GBM patients at Ninewells Hospital) alongside their pre–processing pipeline are also included. The manual tumour segmentation was carried out under the supervision of consultant neurosurgeons, Dr. Kismet Ibrahim (referred here as KHI) and Mr. Mohamed Okasha (referred here as MO). Finally, we describe the multiscale numerical scheme involved in approximating the mathematical model computationally, and present the simulation results as well as discuss future research avenues.

## 2 Materials and methods

This section details the mathematical model that we developed to simulate the evolution of GBM within a three–dimensional fibrous brain environment. Our framework expands the work of Suveges et al. (7) by incorporating the effects of various treatment modalities such as surgery, chemotherapy, and radiotherapy. Furthermore, we postulate our hypothesis and formulate a minimisation problem. Finally, we leverage clinical data from T1, T1+C, T2, and DTI scans to account for factors like brain structure, tumour location and extent, and oedema.

### 2.1 Mathematical multiscale model for GBM progression

#### 2.1.1 Macro–scale dynamics

Following the work from Trucu et al. (3) Suveges et al. (7)Shuttleworth and Trucu (32), we denote by Ω(*t*) the expanding 3–dimensional (3D) tumour region that progresses over the time interval [0*,T*] within a maximal tissue cube *Y* ⊂ℝ^3^. At any macro–scale spatio–temporal point (*x,t*) ∈ *Y* ×[0*,T*], we consider a cancer cell population, denoted by *c*(*x,t*), which interacts with a two–phase heterogeneous ECM (consisting of: a non–fibre *l*(*x,t*) and fibre *F*(*x,t*) ECM phases (7)), while consuming the available nutrients, denoted by *σ*(*x,t*), which are present in the environment. The fibre ECM density, *F*(*x,t*), accounts for all fibrous proteins such as collagen and fibronectin. On the other hand, the non–fibre ECM density, *l*(*x,t*), comprises of non–fibrous proteins (for example, amyloid fibrils), extracellular *Ca* ^2+^ ions, enzymes and polysaccharides (7). Following the methods introduced in Suveges et al. (7), we also incorporate the structure of the brain by extracting data from the modified DTI scan, T1 and T2 brain scans. Finally, we denote by **u**(*x,t*) the global tumour vector which embodies the cancer cell population and the fibre and non–fibre ECM components, given by


$$u\left(x,t\right):={\left(c\left(x,t\right),l\left(x,t\right),F\left(x,t\right)\right)}^{T}.$$

Therefore, the total space occupied by the macroscopic tissue and tumour volume is denoted by *ρ*(**u**) and is defined as


ρ(u)=ρ(c(x,t),l(x,t),F(x,t)):=c(x,t)+l(x,t)+F(x,t),

for all (x,t)∈Ω(t)×[0,T] .

##### 2.1.1.1 Nutrients: *σ(x, t)*

As in this study we focus on avascular tumours, the uptake of nutrients that are available in the outside tissue and are absorbed through the outer tumour boundary plays an important role in the overall tumour development. This nutrients absorption is assumed here to occur at the constant rate *d_σ_ >* 0 and is enabled in the model through the presence of nutrient Dirichlet boundary condition at the evolving tumour boundary *∂*Ω(*t*). Furthermore, the spatio-temporal nutrient transport is assumed to be in diffusion equilibrium, with an autonomous transport diffusion coefficient $D_{\sigma }=D_{\sigma }/\left(c+F+p_{0}\right)$ that takes account of both the presence of the cancer and ECM fibres distributions as well as the baseline permeability *p*_0_ *>* 0 (which is here assumed to be a media constant), while *D_σ_ >* 0 is a constant standing for the maximal diffusive nutrients transport possible in the tissue. Thus, the nutrients dynamics is mathematically given by:


(1) $$\begin{array}{rrr} 0= & \nabla \cdot \left(D_{\sigma }\nabla \right)\sigma -d_{\sigma }c\sigma , & \text{ on Ω}\left(t\right),\forall t\in \left[0,T\right],\\ \sigma \left(x,t\right)= & \sigma_{nor}, & \forall x\in \partial {\text{Ω}}_{0}\left(t\right),\forall t\in \left[0,T\right], \end{array}$$

where *σ_nor_* is the normal level of nutrients in the outside tissue and is considered to be constant, while *∂*Ω_0_(*t*) represents the outside tumour boundary as defined in **Appendix 1** in **Supplementary Material**. Similar to Suveges et al. (33), certain tumour regions become necrotic as soon as the nutrients level *σ* drop below a critical necrotic threshold denoted *σ_n_ >* 0, while *σ_p_ >* 0 represents a nutrient for optimal cancer proliferation regime. Hence, we have the following relationship between these three values: *σ_nor_ > σ_p_ > σ_n_*.

Further, considering here a simpler context than the one in Suveges et al. (33) by focussing only on two nutrient effects (namely, on cell proliferation and cell death rates), we assume that: (1) very low nutrient levels impede cell proliferation (having no proliferation at all in the necrotic regions); and (2) extremely high nutrient levels cannot increase cell proliferation rate by more than a certain maximal proliferation rate Ψ*_p,max_ >* 0 which corresponds to nutrient levels *σ* ≥ *σ_p_*. Thus, mathematically, these two assumptions are accounted for in the modelling via the following nutrient–dependent proliferation function:


(2) $${\text{Ψ}}_{p}\left(\sigma \right):=\{\begin{array}{ll} 0, & \text{if }\sigma \leq \sigma_{n},\\ {\text{Ψ}}_{p,max}, & \text{if }\sigma \geq \sigma_{p},\\ \text{Φ}\left(\sigma ,{\text{Ψ}}_{p,max},0,\sigma_{p}-\sigma_{n}\right), & \text{otherwise}, \end{array}$$

where Φ(σ,.,.,.) describes the smooth transition between the two extrema and is defined to be:


(3) $$\begin{array}{l} \text{Φ}\left(\sigma ,{\text{Φ}}_{max},{\text{Φ}}_{min},{\text{Φ}}_{L}\right):=\\ \frac{\Phi_{max}-\Phi_{min}}{2}\left[cos\left(\frac{\pi \left(\sigma -\sigma_{n}-\Phi_{L}\right)}{\sigma_{p}-\sigma_{n}}\right)+1\right]+{\text{Φ}}_{min}, \end{array}$$

where Φ*_L_* controls the phase shift of the cosine function.

Finally, the effect that the nutrients absence/presence have on cancer cell death is characterised via a function Ψ*_d_* (*σ*) that is of similar type as the one given in Equation 2. Specifically, here we consider a maximal death rate Ψ*_d,max_ >* 0 in necrotic regions, while we assume no death for cancerous cells when the level of nutrients is *σ* ≥ *σ_p_*. Thus, using again the transition function from Equation 3, the effect over the death rate of cancer cells is mathematically expressed as:


(4) $${\text{Ψ}}_{d}\left(\sigma \right):=\{\begin{array}{ll} {\text{Ψ}}_{d,max}, & \text{if }\sigma \leq \sigma_{n},\\ 0, & \text{if }\sigma \geq \sigma_{p},\\ \text{Φ}\left(\sigma ,{\text{Ψ}}_{d,max},0,0\right), & \text{otherwise}. \end{array}$$

##### 2.1.1.2 Cancer cell dynamics: c(x,t)

The spatio–temporal dynamics of the cancer cell population considered in this work accounts for available movement characteristics enabled by T1 and DTI scans (34), based on which the fully anisotropic diffusion tensor, denoted by $D_{T}$ (7, 35–37). In addition to that, the cell population movement is further biased by adhesion processes, which are mathematically captured through a term denoted by $A\left(x,t,\text{u},\theta_{f}\right)$ that will be detailed below. Furthermore, we assume a logistic type proliferation law of the form:


(5) $$P\left(u\right):=\mu {\text{Ψ}}_{p}\left(\sigma \right)c{\left(1-\rho \left(u\right)\right)}^{+},$$

where *µ >* 0 is the proliferation rate regulated by the available nutrients, represented here by the nutrient proliferation function Ψ*_p_* (*σ*) given by Equation 2. Additionally, the term (1 − *ρ*(**u**))^+^ guarantees that we do not experience cell population overcrowding within the available space.

Further, while it is well known that one of the hallmarks of cancer is resisting death (38), nevertheless, due to the abnormal peritumoural vasculature and the degradation of the ECM, nutrient delivery is reduced inside the tumour, ultimately leading to necrosis (33). Therefore, we assume a death rate *d >* 0 that is regulated by the cancer cell death function Ψ*_d_* (*σ*) given by Equation 4. Thus, mathematically the cancer cell death is captured here by the term:


(6) $$Q\left(u\right):=d{\text{Ψ}}_{d}\left(\sigma \right)c.$$

Finally, the population of cancer cells is being reduced further by the effects of chemotherapy and radiotherapy, which are cross–referenced with the patient’s post treatment MRI scans. Hence, the spatio–temporal cancer population dynamics is given mathematically by the following partial differential equation:


(7) $$\begin{array}{l} \frac{\partial c}{\partial t}=\underset{\text{Diffusion}}{\underset{︸}{\nabla \nabla :[D_{T}(x)c]}}-\underset{\text{Adhesion}\;\text{interactions}}{\underset{︸}{\nabla [cA\left(x,t,\text{u},\theta_{f}\right)]}}+P(\text{u})-Q(\text{u})\\ \;-Radiotherapy(c,t)-Chemotherapy(c,t). \end{array}$$

The first term in Equation 7, $\nabla \nabla :\left[D_{T}\left(x\right)c\right]$ , denotes the full second order anisotropic tumour diffusion, with the 3D diffusion tensor $D_{T}$ being constructed from DTI scans of the brain (7, 39). Moreover, the second term in Equation 7, ∇[*c*𝒜(*x,t*,**u** *,θ_f_*)], describes adhesion processes that bias the movement of the cell population due to the adhesion bonds that the migratory cells establish with both the surrounding cell and the ECM components. However, for a compact presentation, we defer the description of both $\nabla \nabla :\left[D_{T}\left(x\right)c\right]$ and $\nabla \left[cA\left(x,t,u,\theta_{f}\right)\right]$ to **Appendix 3** in **Supplementary Material**, where these terms are explained with full details.

The governing equation also accounts for the effects of radiotherapy and chemotherapy. Radiotherapy is administered in multiple sessions scheduled according to five days a week sequence (Monday to Friday) in equal amounts of doses that is captured here mathematically via a subsequence of days {*j_m_*}*_k_*_=1_ *…N_radio_* ⊂ {1*,…,N_final_*} (where {1*,…,N_final_*} represents the entire period of treatment). The intensity of each radiotherapy fraction follows the linear–quadratic model introduced in Bashkirtseva et al. (40) and is delivered here according to an appropriate per-day radiotherapy distribution function $\bar{r}$ : {1*,…,N_radio_*}→ (0,∞), given by $\bar{r}$ (*j_m_*) = *αD*(*j_m_*) + *βD*(*j_m_*)^2^, where *α >* 0 and *β >* 0 are linear and quadratic coefficients of cell damage, and *D*(·): {1*,…,N_radio_*}→ (0,∞) is the per-day radiation dose level distribution (*i.e.*, indicating the dose administered in each scheduled day). Finally, we account here also for the time–overlapping effect of radiotherapy treatment over each time interval $\left(T_{i_{k}}-l,T_{i_{k}}+d\right)$ via the asymmetric mollifier-type function $\psi_{j_{m}}^{radio}\left(t\right)$ , given in **Appendix 5**, **Equation S6** in **Supplementary Material**, ∀*m* ∈{1*,…,N_radio_*}, we have that mathematically the radiotherapy treatment delivery and its effect on the tumour is given by


(8) $$Radiotherapy\left(c\left(x,t\right),t\right):=\sum_{m=1}^{N_{radio}}\bar{r}\left(j_{m}\right)\psi_{j_{m}}^{radio}\left(t\right)c\left(x,t\right).$$

Chemotherapy is incorporated based on the Norton–Simon hypothesis (40), which suggests that tumours are more susceptible to treatment when they have grown for a shorter period of time. Following a chemotherapy scheduling given by a selected subsequence of days {*i_k_*}*_k_*_=1_ *…N_chemo_* ⊂ {1*,…,N_final_*}, we deliver *N_chemo_* doses of chemotherapeutic drug, according to the corresponding per–day chemo agent distribution function *ρ_g_*: {1*,…,N_chemo_*}→ {1,1.1,1.5,2,2.4,2.5,2.8}× *chemo_dose_*, with *chemo_dose_ >* 0 being the initial chemo dose. For this specific patient, the TMZ dose on day 1 is *chemo_dose_* = 130 milligrams, but for example, on day 79, the dose increases to 265 milligrams of TMZ, which results into a corresponding dosage upscaling factor of 265*/*130 ≈ 2. The time–overlapping effect of the chemotherapy over the interval $\left(T_{i_{k}}-l,T_{i_{k}}+d\right)$ is accounted here via a function $\psi_{i_{k}}^{chemo}\left(t\right)$ , given in **Appendix 5**, **Equation S6** in **Supplementary Material**, which is similar in shape to the one for radiotherapy. Further, to account for the fractional cell kill impaired by cytotoxic agent, we adopt an Exponential Kill Model given by *b*(1−*e^ζW^*), where *b >* 0 represents the relative maximum fractional cell kill, *W >* 0 stands for the drug concentration, and *ζ >* 0 describes tumour cells’ sensitivity to the chemo drug. Moreover, the decrease in fractional cell kill as tumour cell population gets closer to its carrying capacity *K >* 0 (representing the maximum cumulative distribution of cells and ECM supported by an infinitesimal volume of tissue) is described here through a Holling type II functional *µK/*(*K* + *sc*), where *µ >* 0 is the growth rate, and *s >* 0 controls the extent of the Norton–Simon effect, *i.e.*, a larger *s* leads to a steeper decline, effectively amplifying the Norton-Simon effect by significantly reducing cell kill effectiveness when the tumour is close to its capacity. Conversely, a smaller *s* results in a more gradual decline, making the Norton-Simon effect less pronounced and allowing for potentially higher cell kill even at larger tumour sizes (40). Thus, chemotherapy delivery and its effect on the tumour is given mathematically by:


(9) $$\begin{array}{l} Chemotherapy\left(c\left(x,t\right),t\right):=\\ \mu b\frac{K}{\left(K+sc\left(x,t\right)\right)}\left(1-e^{\zeta W}\right)\sum_{k=1}^{N_{chemo}}\rho_{g}\left(i_{k}\right)\psi_{i_{k}}^{chemo}\left(t\right)c\left(x,t\right) \end{array}$$

Thus, the governing equation for cancer dynamics finally becomes


(10) $$\begin{array}{l} \frac{\partial c}{\partial t}=\underset{\text{Diffusion}}{\underset{︸}{\nabla \nabla :[D_{T}(x)c]}}-\underset{\text{Adhesion}\;\text{interactions}}{\underset{︸}{\nabla [cA\left(x,t,\text{u},\theta_{f}\right)]}}+P(\text{u})-Q(\text{u})\\ \;-\underset{\text{Radiotherapy}}{\underset{︸}{\sum_{m=1}^{N_{radio}}\bar{r}(j_{m})\psi_{j_{m}}^{radio}(t)c}}-\underset{\text{Chemotherapy}}{\underset{︸}{\mu b\frac{K}{K+sc}(1-e^{\zeta W})\sum_{k=1}^{N_{chemo}}\rho_{g}(i_{k})\psi_{i_{k}}^{chemo}(t)c}}. \end{array}$$

##### 2.1.1.3 Two–Phase ECM macro–scale dynamics: F(x,t) and l(x,t)

The micro–scale mass distribution of fibre ECM phase determines a spatial orientation of ECM fibres at micro–scale level which represents their naturally emerging spatial bias for withstanding incoming cell forces (6). With this orientation, while deferring more consistent details for a later subsection, the ECM fibre phase is therefore represented as a macroscopic vector field *θ_f_* (*x,t*) whose Euclidean norm stands for the amount of fibres at a given macro–scale point (*x,t*), and so *F*(*x,t*): = ∥*θ_f_* (*x,t*)∥_2_ (6, 7). Further, to incorporate the impact of treatment on the each of the two ECM phases, we build on the dynamics of the fibre and non–fibre ECM components introduced in Suveges et al. (7, 33) by considering also the decay effects that the chemo and radio therapies bring about, namely:


(11) $$\frac{\partial F}{\partial t}=-Fc\left(\beta_{F}+\beta_{FChemo}+\beta_{FRadio}\right),$$

(12) $$\frac{\partial l}{\partial t}=-lc\left(\beta_{l}+\beta_{lChemo}+\beta_{lRadio}\right),$$

where *β_FChemo_, β_FRadio_* and *β_lChemo_, β_lRadio_* are the corresponding constant decay rates due to the chemo and radio therapies on the ECM fibres and non-fibres phases, respectively.

##### 2.1.1.4 Summary of the full macro–scale model

In summary, the full model for the macro–scale dynamics is:


(13) $$\{\begin{array}{l} \begin{array}{l} \frac{\partial c}{\partial t}=\underset{\text{Diffusion}}{\underset{︸}{\nabla \nabla :[D_{T}(x)c]}}-\underset{\text{Adhesion}\;\text{interactions}}{\underset{︸}{\nabla [cA\left(x,t,\text{u},\theta_{f}\right)]}}+P(\text{u})-Q(\text{u})\\ \;-\underset{\text{Radiotherapy}}{\underset{︸}{\sum_{m=1}^{N_{radio}}\bar{r}(j_{m})\psi_{j_{m}}^{radio}(t)c}}-\underset{\text{Chemotherapy}}{\underset{︸}{\mu b\frac{K}{K+sc}(1-e^{\zeta W})\sum_{k=1}^{N_{chemo}}\rho_{g}(i_{k})\psi_{i_{k}}^{chemo}(t)c}}, \end{array}\\ \frac{\partial F}{\partial t}=-Fc(\beta_{F}+\beta_{FChemo}+\beta_{FRadio}),\\ \frac{\partial l}{\partial t}=-lc(\beta_{l}+\beta_{lChemo}+\beta_{lRadio}),\\ 0=\nabla \cdot (D_{\sigma }\nabla )\sigma -d_{\sigma }c\sigma , \end{array}$$

in the presence of zero–flux boundary conditions for the cancer, fibre and non–fibre ECM phases, as well as, Dirichlet boundary condition for the nutrients.

#### 2.1.2 Micro–scale dynamics within the bulk and at the tumour boundary

In this section, we focus on the micro–scale processes that contribute to cancer invasion. We first discuss the rearrangement of ECM fibres by cancer cells. ECM fibres are important for providing structural support to tissues. Cancer cells can rearrange ECM fibres using matrix–degrading enzymes (MDEs), such as matrix–metalloproteinases, which allows them to create new pathways for invasion. We then discuss the cell–scale proteolytic process at the edge of the tumour, whereby cancer cells secrete MDEs that degrade the ECM, allowing for further tumour invasion. Finally, we discuss the naturally arising double feedback loop that connects the micro–scale and macro–scale. In this loop, the micro–scale interactions between cancer cells and the ECM influence the macro–scale growth and spread of the tumour. The macro–scale growth and spread of the tumour, in turn, influences the micro–scale interactions between cancer cells and the ECM (6, 7, 33).

##### 2.1.2.1 Micro–scale dynamics of ECM fibres and their macro–scale implications

As described in Shuttleworth and Trucu (6); Suveges et al. (7, 33), the macroscopic ECM fibres alongside their ability to withstand incoming forces are represented through the vector field *θ_f_* (*x,t*) that at each spatio–temporal node (*x,t*) is non–locally induced from their micro–scale configuration, which is given with full details in **Appendix 4** in **Supplementary Material**. This way, the global macro–scale oriented ECM fibre *θ_f_* (*x,t*) characteristics (including its Euclidean magnitude which represent the amount of fibres at (*x,t*), namely *F*(*x,t*):= ∥*θ_f_* (*x,t*)∥_2_, arise and are fully determined from the micro–scale distribution of ECM fibres, providing this way *a fibres bottom–up micro–to–macro scales* link.

However, there exists also *a macro–to–micro scales fibres top–bottom link,* which is triggered by the movement of cancer cells through the ECM fibre distribution that cause the rearrangement of the ECM micro–fibres on each micro–domain *δY*(*x*). Specifically, the fibre rearrangement process is triggered by the macro–scale cancer cell spatial flux


(14) $$ℱ\left(x,t\right):=D_{T}\left(x\right)\nabla c+c\nabla \cdot D_{T}\left(x\right)-cA\left(x,t,\text{u},\theta_{f}\right),$$

which is balanced by the oriented macro–scale ECM fibre *θ_f_* (*x,t*), resulting in a rearrangement flux


(15) $$r\left(\delta Y\left(x\right),t\right):=w\left(x,t\right)ℱ\left(x,t\right)+\left(1-w\left(x,t\right)\right)\theta_{f}\left(x,t\right).$$

with *w*(*x,t*):= *c*(*x,t*)*/*(*c*(*x,t*) + *F*(*x,t*)) being an appropriate mediating weight taking into account the amount of cells transported at (*x,t*) relative to the overall amount of cells and fibres at (*x,t*). This acts uniformly on the mass distribution of micro–fibre on each micro–domain *δY*(*x*), and induces a reallocation of the mass distribution of micro–fibres within both *δY*(*x*) and its adjacent neighbouring micro–domains, as described in Shuttleworth and Trucu (6); Suveges et al. (7, 33).

##### 2.1.2.2 MDEs boundary micro–dynamics and its links to the macro–dynamics

Besides the bulk micro–dynamics that involve the ECM fibres, another key micro–dynamics for tumour invasion is the one involving the proteolytic activity that occurs on the invasive edge of the tumour, enabled by the MDEs (secreted by the cancer cells close to the tumour interface) and transported within the surrounding cell–scale peritumoural tissue neighbourhood. Consequently, this MDE micro–dynamics cause degradation of the peritumoural ECM, thereby inducing alterations in the morphological contours of the tumour boundary (7, 33).

This boundary micro–scale MDEs proteolytic activity is explored via the approach initially introduced in Trucu et al. (3), whereby the emergent spatio–temporal dynamics of MDEs on a micro–scale neighbouring envelope **B**(*∂*Ω(*t*)*,ϵ/*2) of cell–scale thickness *ϵ >* 0, enabled by a bundle *𝒫*(*t*) of overlapping cubic micro–domains $\epsilon Y\left(z\right):=B_{\|\cdot {\|}_{\infty }}\left(z,\epsilon /2\right)$ , ∀ z∈∂Ω(t) , i.e.,


$$𝒫(t):={\left\{\epsilon Y(z)\right\}}_{z\in \partial \Omega (t)}$$

and


$$B\left(\partial \Omega (t),\epsilon /2\right):=\underset{\epsilon Y\in 𝒫(t)}{\cup }\epsilon Y$$

with $B_{\|\cdot {\|}_{\infty }}\left(z,\epsilon /2\right)$ representing the ∥·∥_∞_−ball of radius *ϵ/*2. This facilitates the decomposition of the overarching MDE micro–process occurring on $\cup_{\epsilon Y\in 𝒫(t)}\epsilon Y$ into an assembly of proteolytic micro–dynamics occurring on each distinct *ϵY*. Consequently, at any macroscopic time *t*_0_ ∈ [0*,T*] during the tumour progression, this decomposing bundle *𝒫*(*t*_0_) enable us to explore the MDEs micro–dynamics on each individual micro–domain *ϵY* ∈*𝒫*(*t*_0_), where a source of MDEs emerges naturally at micro–scale on the inner cancer side *ϵY* ∩ Ω(*t*) as result of collective contributions of the macroscopic distribution of cancer cells that arrives during the macro–dynamics within a close proximity, *i.e.*, within distance *γ_h_ >* 0 from *∂*Ω(*t*), which secretes the MDEs. Therefore, mathematically, on a small micro–scale time–length Δ*t >* 0 and at each micro–scale spatio–temporal node (*y,τ*) ∈ *ϵY* × [0,Δ*t*], this source of MDEs induced at the micro–scale by the macro–dynamics is expressed through the non–local term:


(16) $$h(y,\tau )=\{\begin{array}{ll} \frac{\text{B}{\left\|\cdot \right\|}_{\infty }\;{\overset{\int }{\left(y,\gamma_{h}\right)}\text{∩Ω}(t_{0})}^{c(x,t_{0}+\tau )dy}}{\lambda (\text{B}(y,\gamma_{h})\text{∩Ω}(t_{0}))},\; & y\in \text{ϵ}Y\cap \text{Ω}(t_{0}),\\ 0, & y\notin \epsilon Y\backslash (\text{Ω}(t_{0})+\left\{z\in Y{|\left\|z\right\|}_{2}<\rho \right\}) \end{array}$$

where 0 *< ρ < γ_h_* is a small mollification range, **B**(*y,γ_h_*) represents the ∥·∥_∞_ ball of radius *γ_h_* which is centred at a micro–node *y* ∈ *ϵY*. Furthermore, in the presence of this source of MDEs on each of the micro–domains *ϵY*  ∈P (*t*_0_), the MDEs molecular mass–transport across the tumour interface takes place on each *ϵY*. Thus, denoting the MDEs density with *m*(*y,τ*), ∀(*y,τ*) ∈ *ϵY* × [0, Δ*t*], this MDEs transport is assumed here to have a diffusive character and is expressed mathematically as


(17) $$\frac{\partial m}{\partial \tau }=D_{m}\text{Δ}m+h\left(y,\tau \right),\text{ on }\epsilon Y\times \left[0,\text{Δ}t\right],$$

with *D_m_ >* 0 being a constant diffusion coefficient of the MDEs, while this diffusion process is assumed to take place with: (1) null initial conditions, as this is considered to occur with *no molecular memory*; and (2) with flux zero boundary conditions as we assume no MDEs molecular transport across the boundary of *∂ϵY*.

Finally, as this source is induced and determined directly by the macro–scale cancer cell population *c*(·,·), this gives rise to a *top–down* link from the macro–scale to the MDE micro–scale dynamics. On the other hand, as detailed in Trucu et al. (3), the pattern of peritumoural ECM degradation that the MDEs micro–dynamics cause at micro–scale on each boundary micro–domain *ϵY*∈ P (*t*_0_) determines the direction of tumour boundary relocation and enables to characterise this macro–scale movement of the cancer interface through rigorously derived movement laws that specifies precisely at each *x* ∈ *∂*Ω(*t*_0_) the associated relocation direction and magnitude. This ultimately results in a new evolved tumour macro–domain Ω(*t*_0_ + Δ*t*), and this way a *bottom–up* link is established between the boundary MDEs micro–dynamics and the macro–dynamics.

### 2.2 Reconstruction of the cancer–cell distribution within the oedema

**Figure 1:**
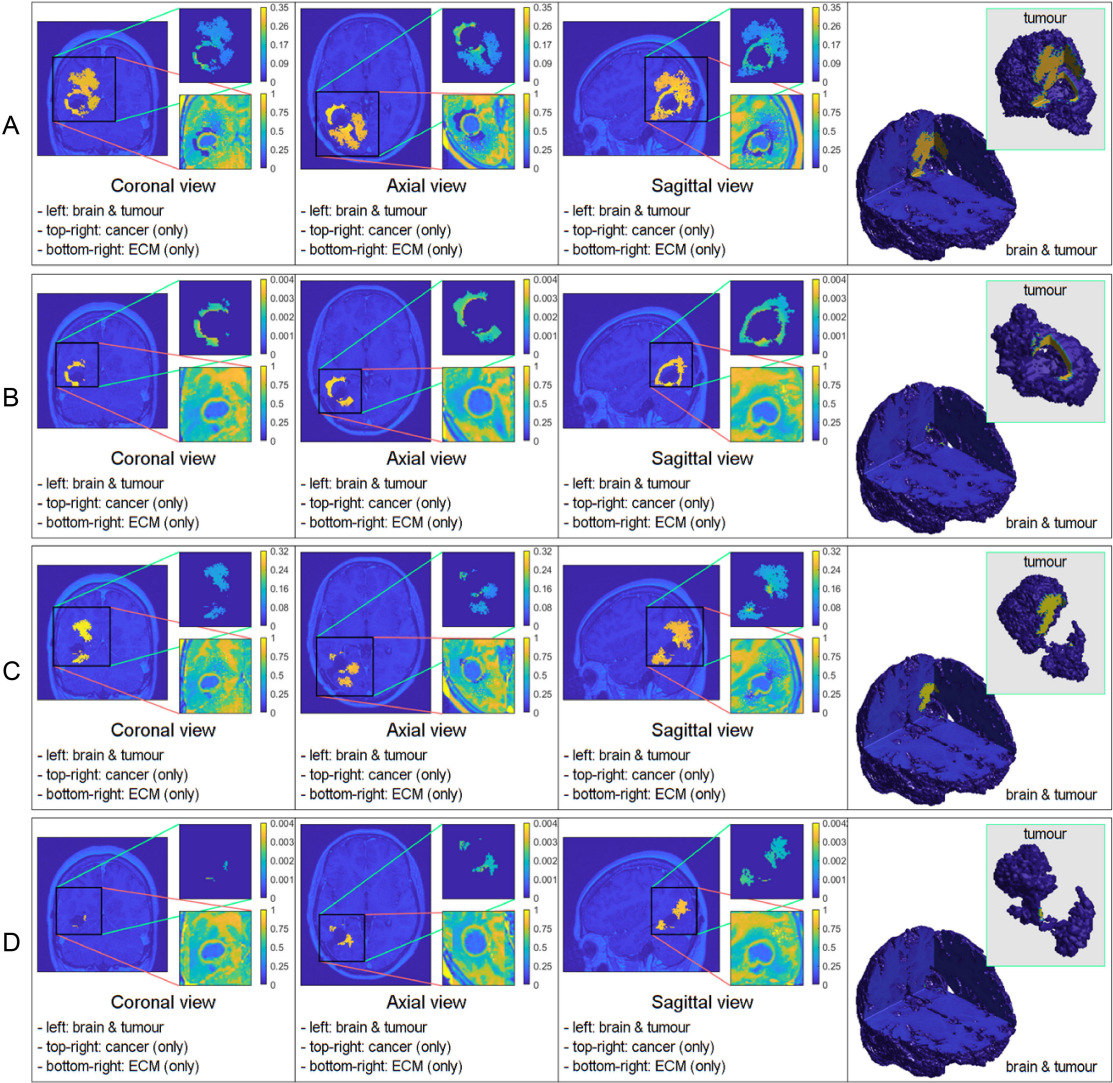
Comparative 3D simulations featuring the mollifier distribution: **(A)** *k_R_* = 5 with no treatment; **(B)** *k_R_* = 5 with treatment; **(C)** *k_R_* = 20 with no treatment; and **(D)** *k_R_* = 20 with treatment. All simulations captured at the final macro–micro stage 45.

**Figure 2:**
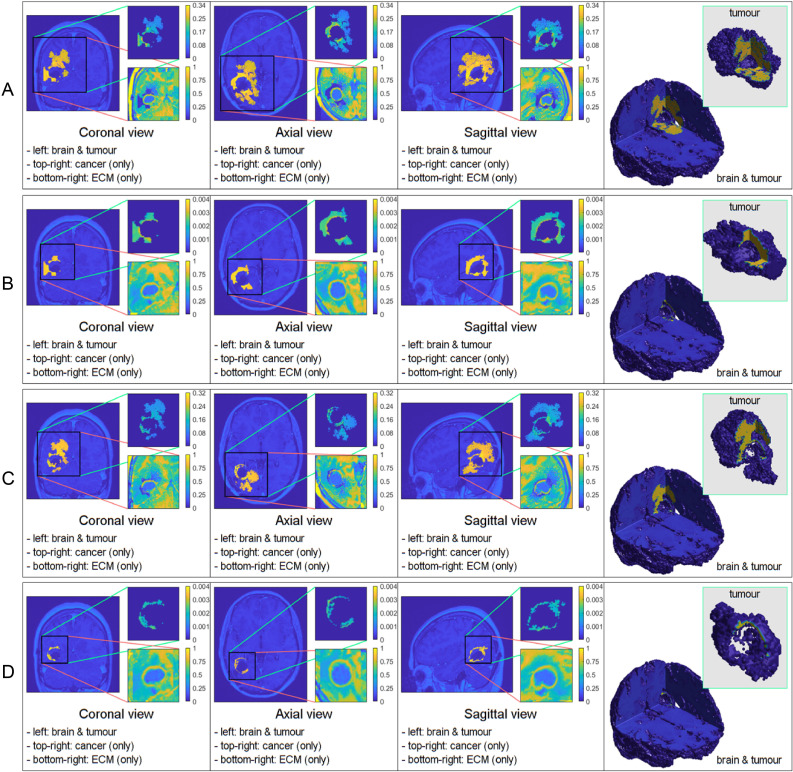
Comparative 3D simulations featuring the Gaussian distribution: **(A)** $k_{\tilde{\sigma }}=10$ with no treatment; **(B)** $k_{\tilde{\sigma }}=10$ with treatment; **(C)** $k_{\tilde{\sigma }}=100$ with no treatment; and **(D)** $k_{\tilde{\sigma }}=100$ with treatment. All simulations captured at the final macro–micro stage 45.

It has been demonstrated that GBM cells invade the surrounding tissue via the peritumoural oedema which is populated by phenotypically distinct cancer cells that persist in the area following surgical intervention (9). While these cells typically remain untreated or survive the chemoradiotherapy treatment, these are not detectable on MRI scans, and contribute to tumour recurrence. Thus, to gain a better understanding of the tumour relapse process after surgery, it is of interest to explore whether there is any correlation between the shape of the distribution of GBM cells that remain within oedema right after surgery and the extent of the subsequent tumour relapse. Several numerical experiments that we carried out (as those shown in **Figures 1**, **2**) suggest the following hypothesis, namely:

**H:** *a distribution of GBM cells within the oedema that has most cells mass concentrated within the immediate proximity of the cavity edge leads to a more limited spread and a slower progression of the tumour relapse.*

This hypothesis also aligns with clinical findings suggesting that surgical resection removes a substantial number of cancer cells, leaving the remaining cells more dispersed throughout the oedema (41, 42).

In the following, hypothesis **H** will be examined on two relevant oedema cancer cell distribution types. Furthermore, in both cases, we propose a clinical data assimilation approach, by which we aim to reconstruct the particular shape of the cancer cell distribution that enables the predictive computational modelling solution for the post–surgery GBM relapse to match the available MRI imaging data.

**Figure 3:**
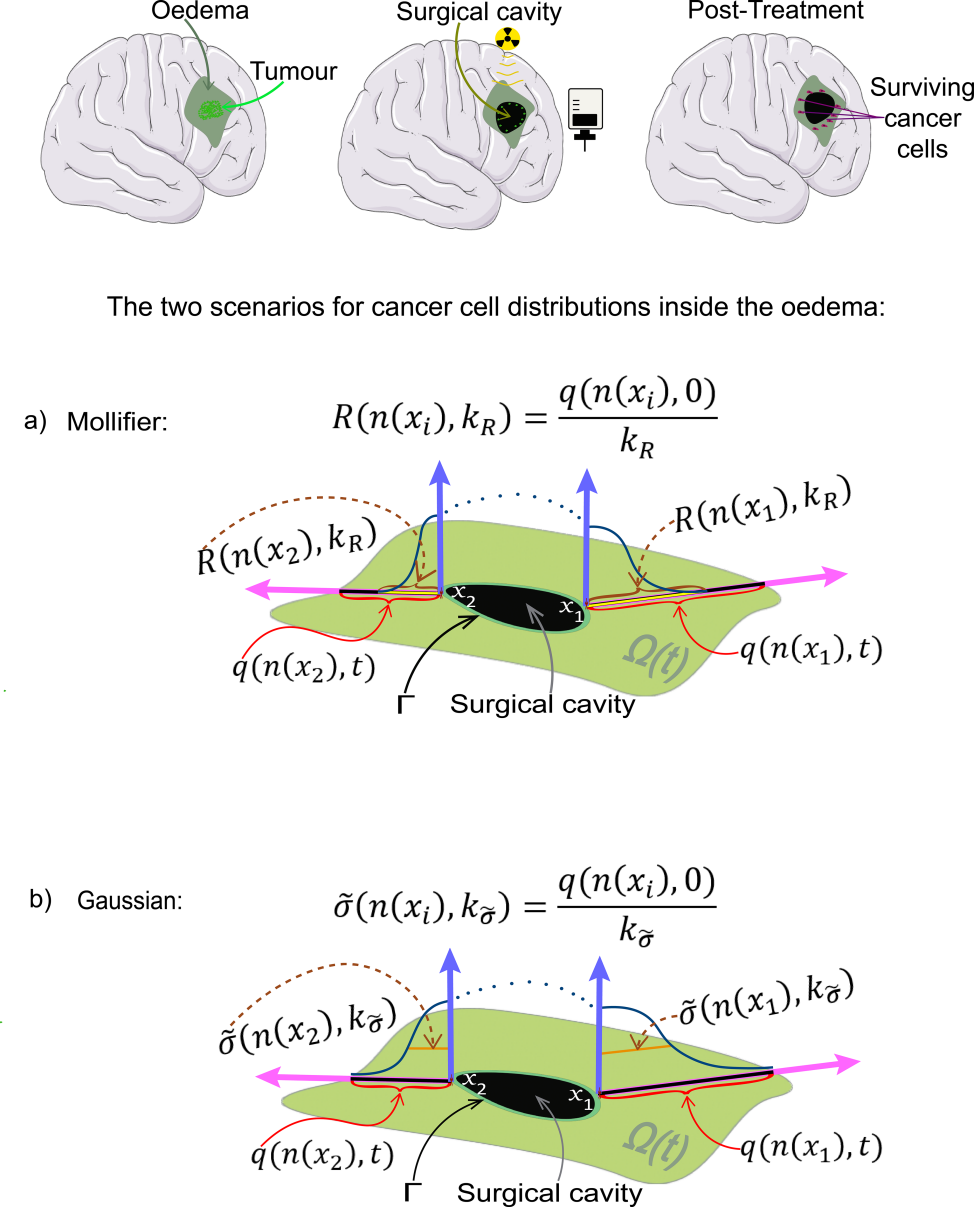
Schematic showing from top right to bottom left: a GBM tumour, radio and chemotherapy being applied to the surgically removed area, the surviving cells inside the oedema and finally the two scenarios of cancer cell distributions, **(A)** the mollifier, and **(B)** the Gaussian distributions, used in the simulations, where Γ represents the edge of the surgical cavity.

***Two possible post–surgery oedema cancer cell distribution scenarios:*** In the following, we explore hypothesis **H** by considering two possible scenarios for the post–surgery oedema cancer cell spatial distribution, namely one that is compactly supported strictly within Ω(0) and one that carries non–zero cell mass density distributed at any point in Ω(0). Specifically, denoting by *n*(*x*) the usual outward unit normal vector to the surgical cavity edge Γ, ∀*x* ∈ Γ, we assume that:

on the positive side of the normal direction associated to any *x* ∈ Γ, represented here parametrically by


$$d_{x}:=x+\upsilon n(x),\;\upsilon \geq 0,$$

the shape of immediate post–surgery cancer cell distribution remaining within the oedema along *d_x_*, denoted here by $c_{oedema}^{d_{x}}$ , is of either of the following two types:

case 1: a smooth compact support mollifier–type distribution of support radius $R\left(n\left(x\right),k_{R}\right)$ centred at *x*, which is given by


$$c_{oedema}^{d_{x}}\left(\upsilon \right):=R{\left(n\left(x\right),k_{R}\right)}^{-1}\psi_{1}\left(\frac{\upsilon }{R\left(n\left(x\right),k_{R}\right)}\right),\;\upsilon \in \left[0,q\left(n\left(x\right),0\right)\right],$$

where *ψ*_1_(·) is the 1D standard symmetric mollifier given in **Appendix 5** in **Supplementary Material**, while, for any *t* ≥ 0, *q*(*n*(*x*)*,t*) denotes the distance along line *d_x_* between Γ and *∂*Ω(*t*), with $R\left(n\left(x\right),k_{R}\right):=\frac{q\left(n\left(x\right),0\right)}{k_{R}}$ while $k_{R}>1$ represents an uniform scaling constant applied at each *x* ∈ Γ controls the cancer cells distribution spread in the normal direction described by *n*(*x*), see **Figure 3A**);

case 2: a Gaussian distribution centred at *x* and of standard deviation $\tilde{\sigma }(n\left(x\right),k_{\tilde{\sigma }}$ ), which is given by


$$c_{oedema}^{d_{x}}\left(\upsilon \right)\propto N_{d_{x}}\left(0,\tilde{\sigma }\left(n\left(x\right),k_{\tilde{\sigma }}\right)\right),\;\upsilon \in [0,q\left(n\left(x\right),0\right)],$$

where by $N_{d_{x}}\left(0,\tilde{\sigma }\left(n\left(x\right),k_{\tilde{\sigma }}\right)\right)$ we denote here the family of normal distributions along *d_x_*, with $\tilde{\sigma }\left(n\left(x\right),k_{\tilde{\sigma }}\right):=\frac{q\left(n\left(x\right),0\right)}{k_{\tilde{\sigma }}}$ , while $k_{\tilde{\sigma }}>1$ represents an uniform scaling constant applied at each *x* ∈ Γ controls the standard deviation, see **Figure 3B**).

**Figure 4:**
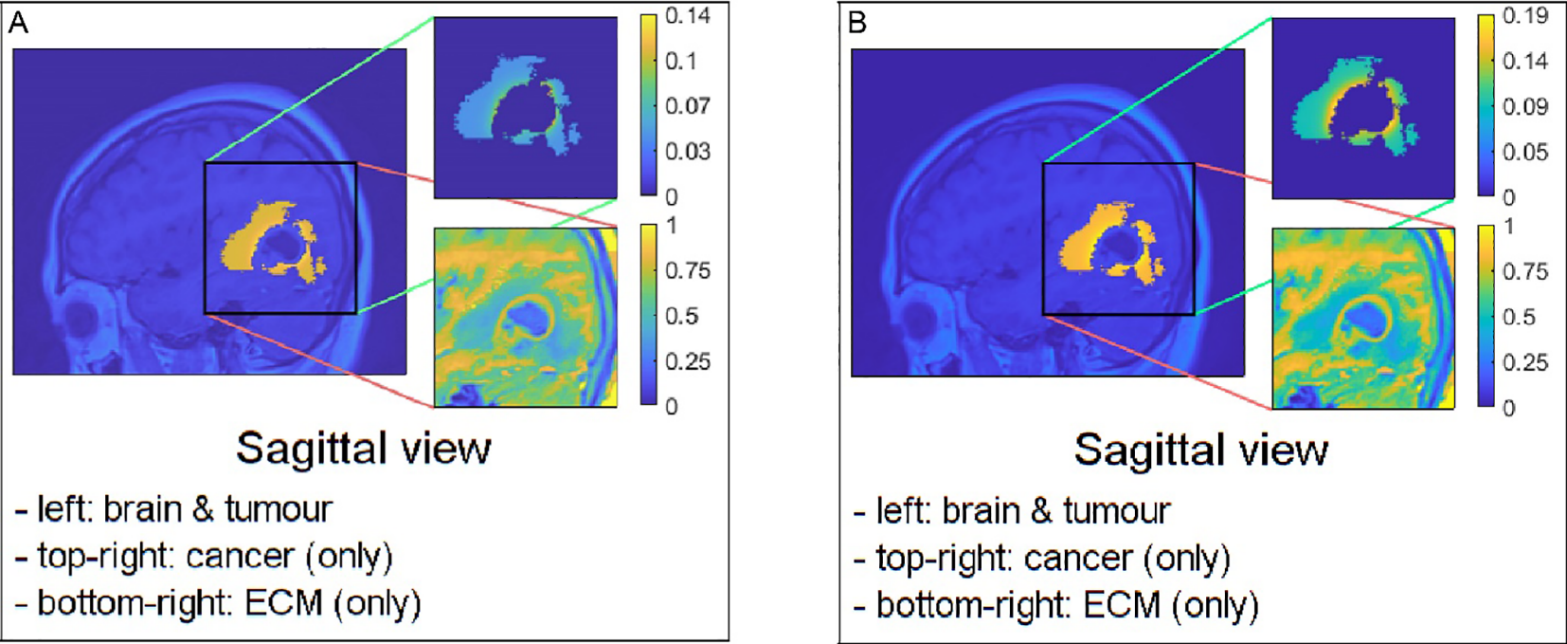
Sagittal views of possible examples of initial conditions when applying them to the pre–surgical oedema: **(A)** the mollifier and **(B)** the Gaussian distribution.

For each of the two cases, we explore the correlation between the extent of significant tumour spread within oedema (characterised in case 1 by $R\left(n\left(x\right),k_{R}\right)$ and in case 2 by $\tilde{\sigma }\left(n\left(x\right),k_{\tilde{\sigma }}\right)$ ) and the extent of tumour invasion post–surgery. A smaller $R\left(n\left(x\right),k_{R}\right)$ and $\tilde{\sigma }\left(n\left(x\right),k_{\tilde{\sigma }}\right)$ corresponds to a higher concentration of cells near the cavity’s edge, with density decreasing as we move further away from it, as evident in **Figure 3** and the upper–right image of **Figure 4**. Finally, we take advantage of available MRI scans to identify suitable values for $R\left(n\left(x\right),k_{R}\right)$ and $\tilde{\sigma }\left(n\left(x\right),k_{\tilde{\sigma }}\right)$ that enable the closest possible match between the computed solutions and the imaging data.

***Reformulation as least square minimisation problem*:** In order to assimilate available MRI data to identify appropriate values of parameters controlling the degree of spread of the residual cancer cells within oedema (namely, $R\left(n\left(x\right),k_{R}\right)$ and $\tilde{\sigma }\left(n\left(x\right),k_{\tilde{\sigma }}\right)$ for case 1 and case 2, respectively), we proceed by conceptualising this as a minimisation problem. Indeed, to achieve this, to address simultaneously both cases, we consider the mapping *Z*(*n*(*x*),·): (1,∞) → (0*,q*(*n*(*x*),0)) that is defined at each *ξ* ∈ (1,∞) by


(18) $$Z\left(n\left(x\right),\xi \right):=\{\begin{array}{ll} R\left(n\left(x\right),\xi \right), & \text{ for case }1\\ \tilde{\sigma }\left(n\left(x\right),\xi \right), & \text{ for case }2 \end{array}$$

with $R\left(n\left(x\right),\xi \right):=\frac{q\left(n\left(x\right),0\right)}{\xi }$ in case 1, and $\tilde{\sigma }\left(n\left(x\right),\xi \right):=\frac{q\left(n\left(x\right),0\right)}{\xi }$ in case 2. In this context we aim to identify the point of minimum $\xi_{min}$ (representing the optimal controller parameters ${\bar{k}}_{R}$ and ${\bar{k}}_{\tilde{\sigma }}$ ) in case 1 and case 2, respectively) that minimises the following ξ− dependant distance


(19) $$dist\left(c_{Z\left(n\left(x\right),\xi \right)},MRI\right):=\underset{i=1,\ldots ,N_{data}}{\max}\|c_{Z\left(n\left(x\right),\xi \right)}\left(\cdot ,t_{i}\right)-MRI_{i}{\|}_{2},$$

where ${\left\{t_{i}\right\}}_{i=1,\ldots ,N_{data}}$ are the macroscopic times at which the corresponding *MRI* scans ${\left\{MRI_{i}\right\}}_{i=1,\ldots ,N_{data}}$ will have been recorded. Moreover, *MRI_i_* is the previously obtained *“volume of interest”* (VOI). Here, $c_{Z\left(n\left(x\right),\xi \right)}\left(\cdot ,t_{i}\right)$ represents the spatial density of the computed solution evaluated at *t_i_* that is obtained for a *guessed initial condition* $c_{0}^{guess}\left(\xi ;d_{x},\upsilon \right)$ that corresponds to ξ∈(1,∞) . Finally, for each ξ∈(1,∞) , the guessed initial condition $c_{0}^{guess}\left(\xi ;d_{x},\upsilon \right)$ is defined in each of the two cases as:

**c**ase 1: $c_{0}^{guess}\left(\xi ;d_{x},\upsilon \right):=R{\left(n\left(x\right),\xi \right)}^{-1}\psi_{1}\left(\frac{\upsilon }{R\left(n\left(x\right),\xi \right)}\right)$ ,  υ∈[0,q(n(x),0)] ,

**c**ase 2: $c_{0}^{guess}\left(\xi ;d_{x},\upsilon \right)\propto N_{d_{x}}\left(0,\tilde{\sigma }\left(n\left(x\right),\xi \right)\right)$ ,  υ∈[0,q(n(x),0)] .

### 2.3 Clinical data assimilation

#### 2.3.1 Acquisition of clinical data

The clinical data used for this study was acquired from one single GBM patient who received different treatments at Ninewells Hospital between 2017 and 2021, chosen due to their prolonged survival, giving us access to multiple MRI scans which can be used to improve our mathematical model. Ethical approval was obtained from the local Caldicott Guardian, Integrated Research Application System (IRAS)(project ID: 309957), Tayside Research and Development Committee (project ID: 2022NH01) and Research Ethics Committee (REC) (Ref: 22/NS/0021). To be included in the study, patients had to be over 16 years old but no older than 85 years old, with histologically confirmed GBM, and have undergone multiple pre–operative and post–operative MRI scans and received standard NHS chemotherapy and radiotherapy treatments. Patients with a limited number of MRI scans were excluded.

#### 2.3.2 Brain imaging, preprocessing and segmentation

The MRI scans were conducted using NHS GE 1.5 Tesla scanners and included multiple pre–operative and post–operative scans for the selected patient. The scans consisted of T1–weighted (T1), T2–weighted (T2), contrast–enhanced T1–weighted (with Gadolinium) (T1+C), diffusion–weighted imaging (DWI) (for specific dates), and T2–FLAIR sequences.

**Figure 5:**
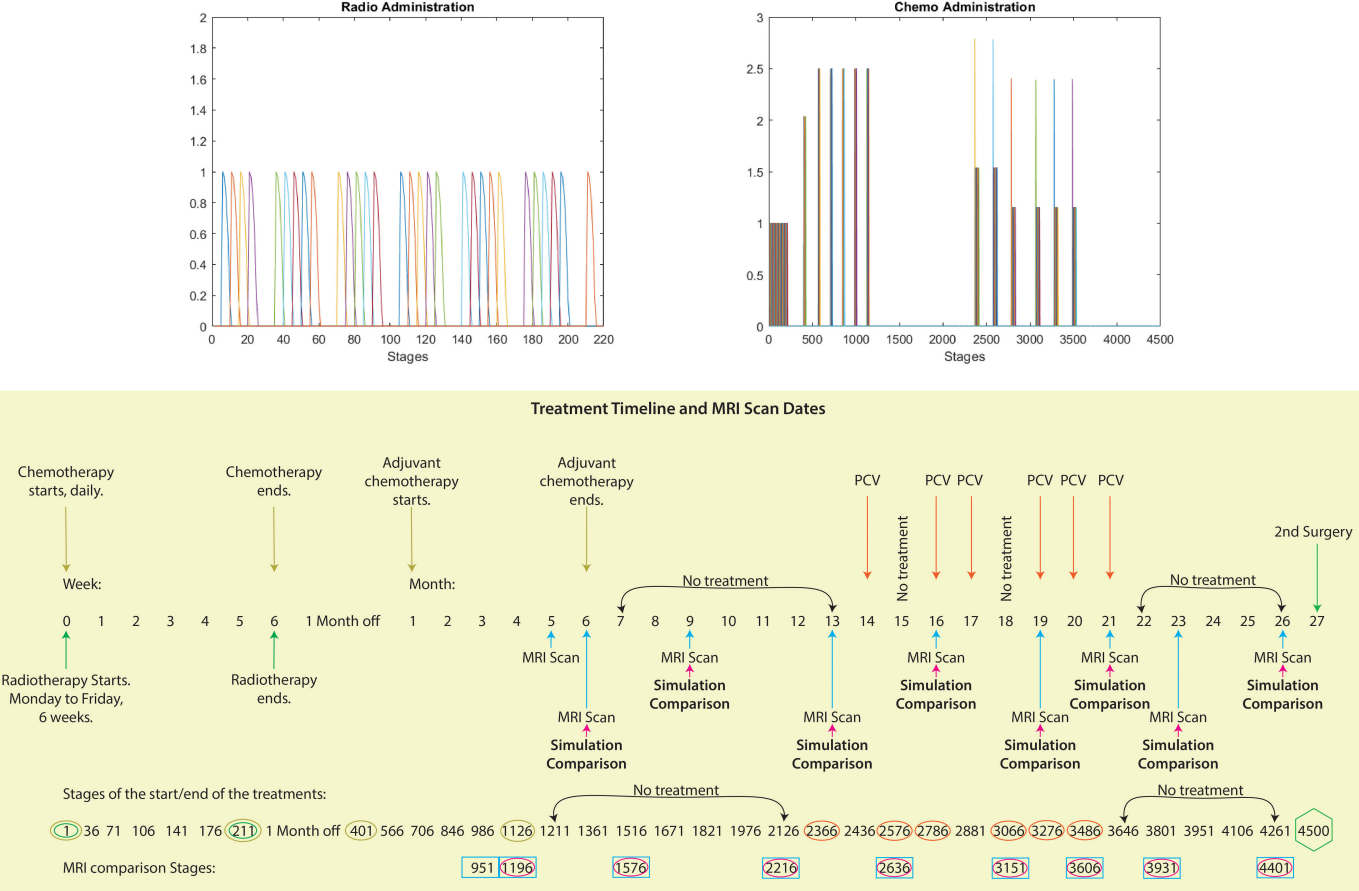
Visualising treatment dynamics: Top image: These graphs depict the radiotherapy and chemotherapy delivery for this patient. The horizontal axis represents the computational stages of the treatment simulation, with every five stages corresponding to one actual day. The vertical axis represents the intensity of the radiation and chemotherapy doses delivered at each stage. Bottom image: Schematic showcasing the full timeline between the first and second surgery from the case considered of the treatments administered (radiotherapy, TMZ, adjuvant TMZ and PCV), the MRI scans dates and the corresponding dates of the comparison between our simulations and the MRI scans. Moreover, we have aligned the temporal progression (weeks and months) of the treatment phases with the corresponding stages of our computational model, providing a clear overview of the timeline and key milestones.

A single typical patient from the series was used for the calculations described here. The patient received initial surgery, followed by chemoradiotherapy with Temozolomide (TMZ) at 130 mg per day concurrently with radiotherapy at a total of 60 Gy distributed equally in 30 total fractions, following the Stupp protocol, and in addition, adjuvant TMZ at a dose of 265–325 mg (6 cycles) after the initial radiotherapy and chemotherapy treatments. Due to recurrence, visible seven months after the completion of concurrent chemoradiotherapy and adjuvant TMZ, the patient also received Lomustine at 160 mg, Procarbazine at 150–200 mg, and Vincristine at 1–2 mg (6 cycles) (this treatment is named PCV). The delivery of the radiotherapy and chemotherapy can be seen in **Figure 5**, where we also considered all chemotherapeutic drugs functionally equivalent (TMZ, Lomustine, Procarbazine and Vincristine), adjusting only the dosage based on the treatment plan. With the purpose to simulate this treatment delivery, we need to modify the model such that every computational macro–micro stage corresponds to a certain amount of real time. This is later explained in Section 3.1, but clearly showcased in the bottom schematic of **Figure 5**.

The patient underwent two more surgeries, and MRI scans were taken before and after each of the surgeries, as well as after the completion of the different treatments. When the patient was not undergoing any treatments, MRI scans were conducted every three to four months.

The MRI scans were first pre–processed using Statistical Parametric Mapping (SPM–12, http://www.fil.ion.ucl.ac.uk/spm/). This pre–processing involved reslicing, normalising and finally segmentation of the T1 scan to obtain the white and grey matter densities. As Diffusion Tensor Imaging (DTI) scans were not obtained for this patient, we modified a standard DTI scan from a healthy volunteer from the IXI Dataset (http://brain-development.org/ixi-dataset/), which was warped to match the anatomy of the T1 scan of the GBM patient, hence we were able to infer brain fibre tract directions for the GBM brain.

**Figure 6:**
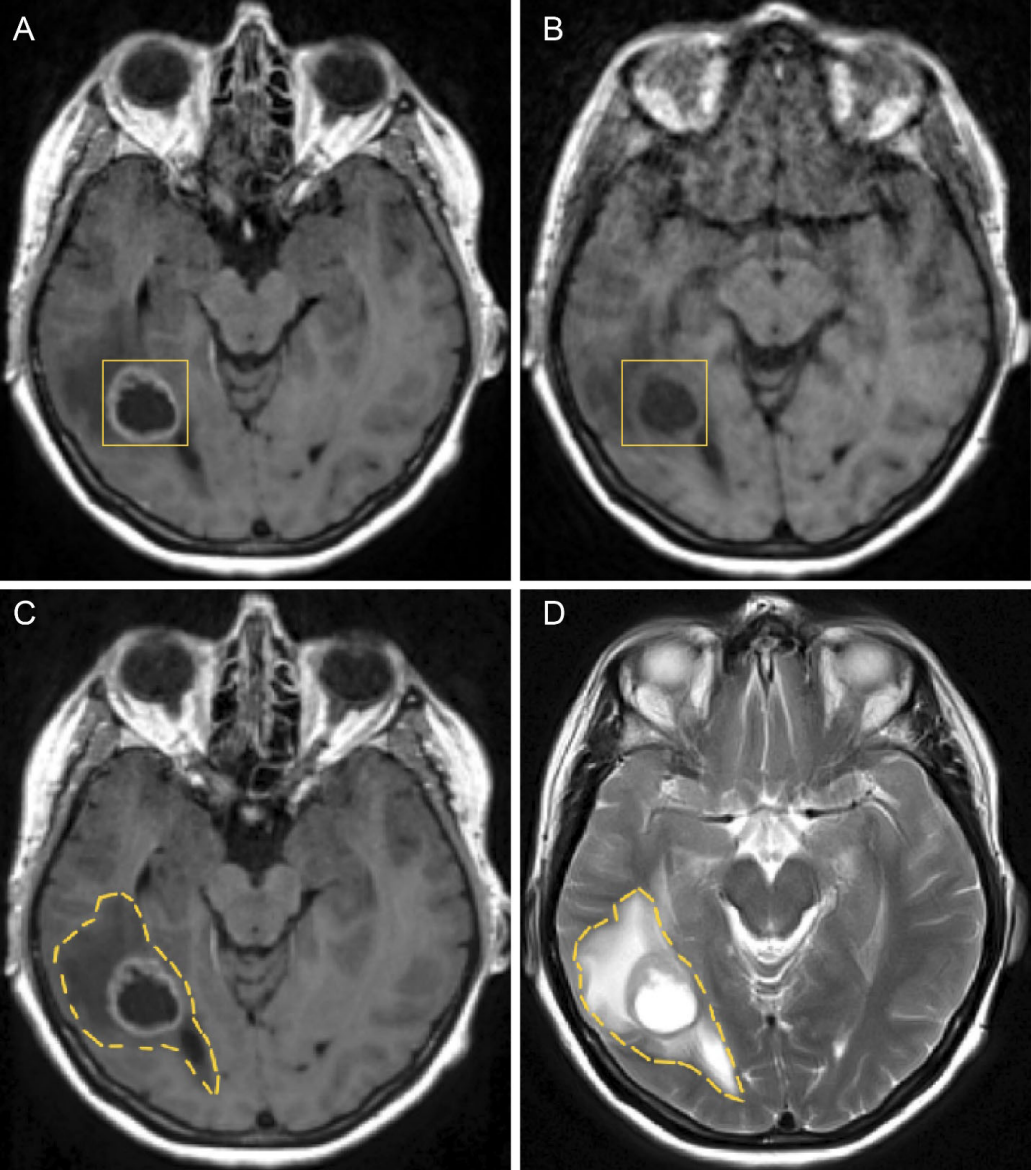
Figure showing GBM and oedema in different axial MRI scans before the first surgery: **(A)** GBM tumour in a T1+C, **(B)** GBM tumour in a T1 without contrast, **(C)** oedema in a T1+C and **(D)** oedema in a T2 weighted scan.

In **Figure 6**, on the top right, a T1–weighted scan (T1) is shown and on the top left a T1 scan with gadolinium contrast (T1+C), which outlines the tumour as gadolinium is taken up by the invasive edge of the tumour. The GBM proliferating edge is observed as enhancing in the T1+C and hypo- to iso- intense in T1, as seen in **Figure 6**. The region which is hyperintense in the T2 scan (and T2–FLAIR) and it is non–enhancing in T1+gadolinium represents the oedema, as seen in the bottom row of **Figure 6**.

**Figure 7:**
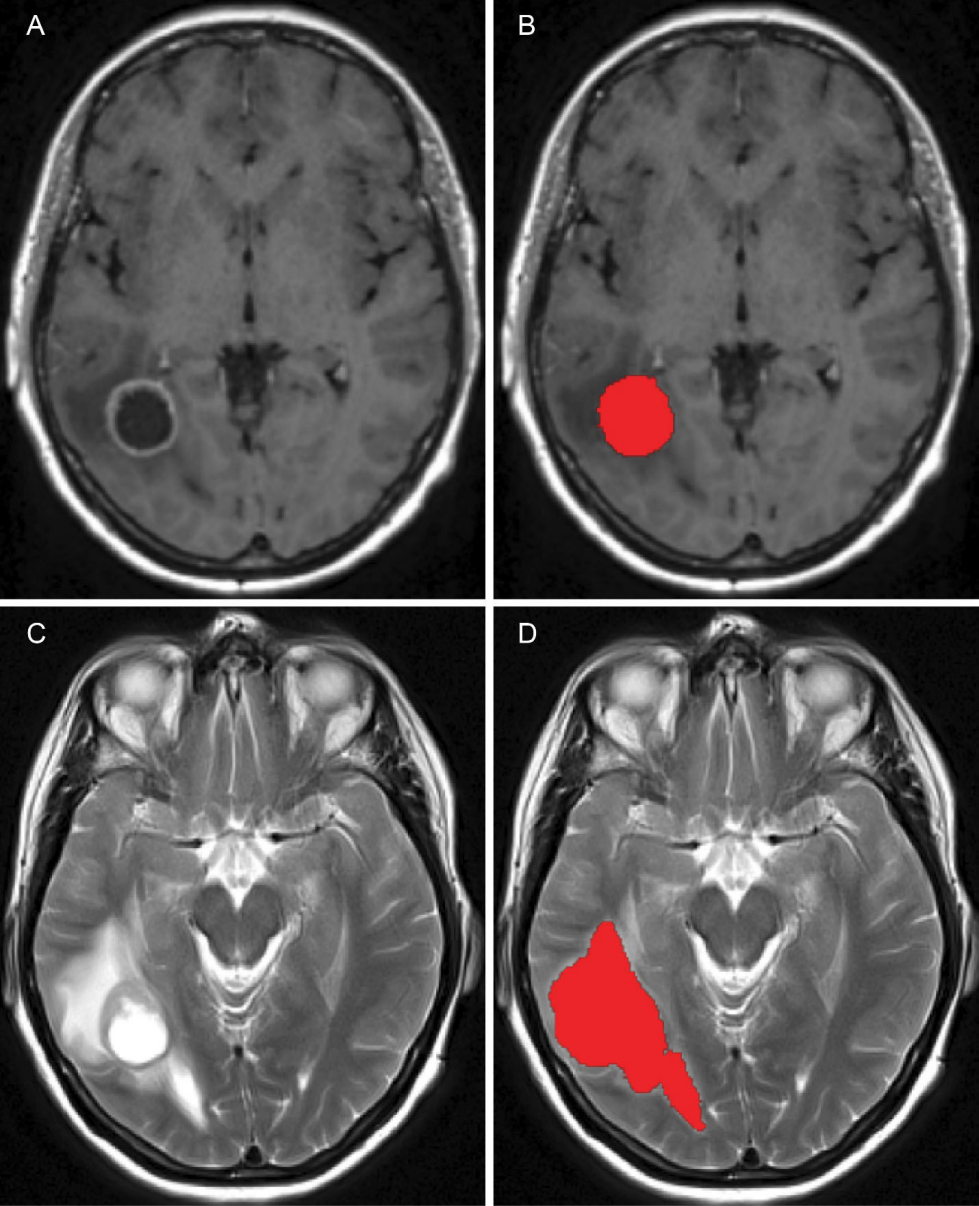
Figure showing the pre–surgical axial MRI scans and corresponding volumes of interest for the patient in question: **(A)** T1+C scan, **(B)** Pre–surgical GBM VOI, **(C)** T2 scan and **(D)** oedema VOI.

After pre–processing has been completed, tumour segmentation was done using MRIcroGL, version v1.2.20220720 (www.nitrc.org). The segmentation was performed manually, under the supervision of NHS Consultant neurosurgeons KHI and MO, who specialise in the treatment of GBM. The scans were processed on a axial (transverse) slice–by–slice basis, as seen in **Figure 7**, for the post–contrast T1 and T2 sequences. These enabled the exploration of important characteristics, referred to as *“volumes of interest”* (or simply “VOI”), one for the pre–surgical tumour and another one for the oedema before surgery, which were given in the form of binary masks (*i.e.*, individual indicator matrices of zeros and ones that give the footprints of the tumour and oedema) and that were later used in our mathematical model.

## 3 Results

The numerical approach employed in this work to tackle both the macro–scale and micro–scale dynamics, as well as the top–down and bottom up links between the scales, builds on a sequence of multiscale modelling and computational works introduced in Trucu et al. (3); Shuttleworth and Trucu (6, 32); Suveges et al. (7, 33), and extends these through the introduction of a new governing equation for capturing nutrients dynamics. Moreover, to identify the shape of the remaining post–surgery oedema cancer cell population distribution that lead to GBM relapse, the 3D computational modelling platform developed here is coupled with a least–square–type clinical data assimilation approach using post–surgical MRI scans.

Similar to the methodology outlined in Suveges et al. (33), we utilise the successive over–relaxation method for solving the nutrients Equation 1. For the rest of the macro–scale dynamics in (13), we follow similar steps as in Suveges et al. (7, 33) and employ the method of lines with the following details. Specifically, the spatial operators (*i.e.*, the diffusion and adhesion operators) are addressed as follows: (a) for diffusion we implement a symmetric finite difference scheme based on convolution, as detailed in Suveges et al. (33); and (b) for adhesion we utilise a convolution–driven approach employing a *fifth–order weighted essentially non–oscillatory* (WENO5) finite difference scheme (43–46), also elaborated upon in Suveges et al. (33). Finally, the time marching is ensured through a predictor corrector scheme introduced in Shuttleworth and Trucu (6) and further detailed in Suveges et al. (7, 33).

### 3.1 Treatment scheduling

One of the primary aims of our work is to accurately replicate the treatment regimen and dosages administered to a specific patient, which in this case, revolves around the time span bridging the first and second surgery, during which various treatment modalities were employed throughout this entire duration.

The comprehensive timeline for this patient extends to 900 days, encompassing the period between the first and second surgery. During this span, chemotherapy and radiotherapy were administered, and MRI scans were conducted on specific dates, as shown in the bottom schematic of **Figure 5**. In order to forecast the possibility of relapse and tumour spread based on this patient’s treatment timeline, we need to simulate the treatment process over the course of these 900 days. To achieve this goal, it is important to demarcate the computational macro–micro stages and steps meticulously.

To precisely capture the daily dynamics of the patient’s treatment, the model was modified with a temporal discretisation scheme. Here, every five computational stages represent one actual day, resulting in 4500 stages. This discretisation comes from splitting the macro–scale time interval (*i.e.*, the treatment duration) [0*,T_f_*] into smaller intervals {[*k*Δ*t*, (*k*+1)Δ*t*]}*_k_*_=0,_ *k_max_*. Each such increment, which encompasses both the macro–dynamics that takes place on Ω(*k*Δ*t*) over the time period [*k*Δ*t*, (*k*+1)Δ*t*] and the micro–dynamics at its boundary (influenced by the “top–down” links (explained previously in greater detail in section 2.1.2) on each of the boundary micro–domains ϵ*Y* ∈ **B**(*∂*Ω(*k*Δ*t*),ϵ*/*2)) constitutes a *“stage k”*. As described in Trucu et al. (3)Alzahrani et al. (47), these micro–dynamics at the boundary dictate the precise direction and displacement magnitude for the relocation of each of the points on *∂*Ω(*k*Δ*t*)), progressing this way the stage k tumour domain Ω(*k*Δ*t*) into the newly obtained domain Ω((*k* + 1)Δ*t*). With this method, we can match the exact treatment for each day and compare our simulations with the MRI scans taken on those specific dates.

### 3.2 Initial conditions

The initial micro–fibre distribution within a micro–domain *δY* (*x*) is considered here to be the one introduced in Suveges et al. (7), which in brief can be summarised as follows. On one hand, if *x* ∈ *Y* is located in the grey matter zone, random straight narrow 3D–stripes (*i.e.*, narrow equal–square cross–section parallelepipedic bars that fit within *δY* (*x*)) are distributed until the ratio of the cumulative stripe volume occupied 35% out of the entire *δY* (*x*). On the other hand, if *x* is located in the white matter, a predefined set of aligned straight narrow 3D–stripes is distributed within *δY* (*x*) until the volume is filled up to the same percentage, *i.e.*, up to 35%. We also incorporated information about the white and grey matter tracts from the T1+C scan into the micro–scale fibre distribution (7). For the non–fibre ECM phase, we have the following initial condition:


(20) $$l\left(x,0\right)=\text{min }\{h\left(x_{1},x_{2},x_{3}\right),1-c\left(x,0\right)\},$$

where for any $x:=\left(x_{1},x_{2},x_{3}\right)\in Y$ we have:


$$h\left(x\right)=\frac{1}{2}+\frac{1}{4}\text{sin }{\left(7\pi y_{1}\left(x\right)y_{2}\left(x\right)y_{3}\left(x\right)\right)}^{3}\cdot \text{sin }(7\pi y_{1}\left(x\right)/y_{2}\left(x\right)/y_{3}\left(x\right)),$$

with:


$$\begin{array}{lll} y_{1}\left(x\right) & := & \frac{1}{3}\left(x_{1}+1.5\right),\\ y_{2}\left(x\right) & := & \frac{1}{3}\left(x_{2}+1.5\right),\\ y_{3}\left(x\right) & := & \frac{1}{3}\left(x_{3}+1.5\right). \end{array}$$

Lastly, the initial condition for the nutrients is set to: *σ*(*x*,0) = 0.4.

### 3.3 Numerical simulations

This section presents the results of 3D numerical simulations of the multiscale model of GBM tumour growth. The parameter values used in the simulations are taken from **Table S1** in **Supplementary Material**. Any modifications made to the values are stated in the text.

To display the evolution of the tumours at time 45Δ*t*, we show four panels for each simulation. The first three panels show the tumour in the coronal, axial, and sagittal planes, respectively. The final panel shows a 3D image of the brain with the embedded tumour alongside the 3D tumour in isolation.

The figures below show the evolution of GBM tumours with different cancer cell distributions in the oedema, under the application (or not) of radiotherapy and chemotherapy. The densities of the main tumour and the ECM are shown in the top–right and bottom–right corners of each of the three classical–views panels, respectively.

Now, to initialise our simulations, we use the segmented masks for both the pre–surgical oedema and tumour, which are subtracted in order to create a surgical cavity, as depicted in **Figure 3**. Next, we apply either a cancer cell distribution within the modified oedema mask of the shape of a mollifier–type distribution or a Gaussian–type distribution. Moreover, the treatment used on this specific patient is also being applied at the simulation, as shown in **Figure 5**.

**Figure 8:**
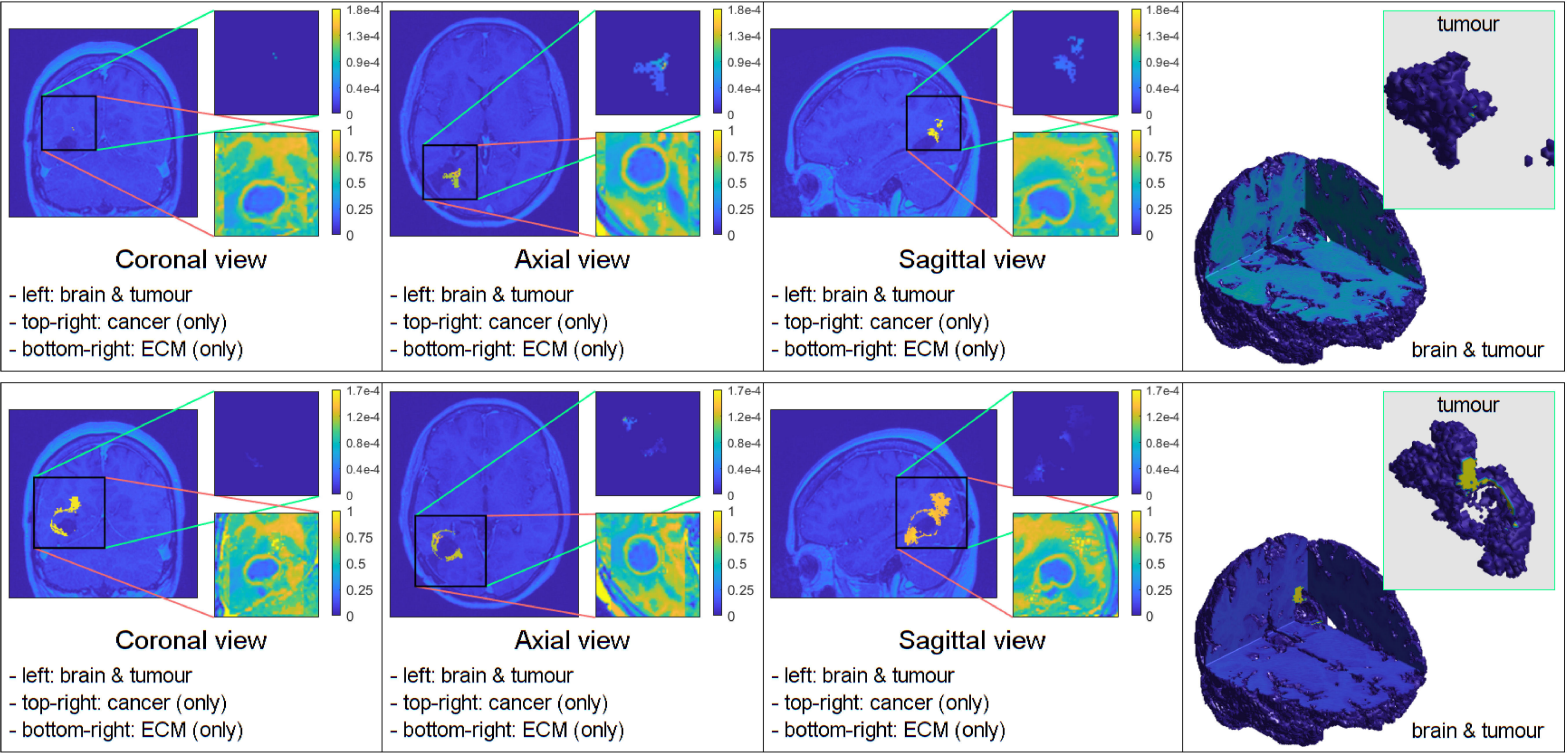
Simulations that showed the least tumour progression with the mollifier (top) and Gaussian distribution (bottom), respectively.

The figures below show the results of applying the mollifier distribution with different values for $k_{R}$ , in **Figure 1** and the Gaussian distribution with different values for $k_{\tilde{\sigma }}$ , in **Figure 2**. Finally, we compare the results which showed a reduction in tumour size, as shown in **Figure 8**.

The results of the simulations are consistent with clinical data, which have shown that the highest concentration of cancer cells in recurrent GBM patients are located at the resection margin (41, 42), hence using the oedema mask, and applying either a mollifier or Gaussian distribution of cancer cells within it, can lead to clinically relevant results with the appropriate values for $k_{R}$ or $k_{\tilde{\sigma }}$ , respectively.

**Figure 1** illustrates the results from two experiments. In the first experiment, rows A) and B) used the parameter value of *k_R_* = 5. Row A) depicts the results obtained without applying any treatment, whilst row B) shows the simulation when the treatment from **Figure 5** was applied throughout the macro–micro stages.

The second experiment, showcased in rows C) and D), used *k_R_* = 20. Similarly to the first experiment, row C) presents the results without any treatment, while row D) showcases the simulation with the treatment applied. Observe that applying the treatment, in **Figure 1** rows B) and D), it highly reduces the densities and spread of the tumour, but there are still residual cancer cells left, mostly around the surgical cavity. Finally, observe that increasing the value of $k_{R}$ leads to less spread, when comparing the top two rows (A and B) with the bottom two (C and D).

Similarly to the previous case, **Figure 2** displays the simulations using the Gaussian distribution with no treatment being applied in rows A) and C), whilst rows B) and D) are the simulations with chemoradiotherapy. Moreover, a value of $k_{\tilde{\sigma }}=10$ is used for both rows A) and B) and $k_{\tilde{\sigma }}$ = 100 for both rows C) and D). When using the Gaussian distribution for the residual cancer cells within oedema after surgery, we observe a similar morphology to the previous case. As with the mollifier distribution experiment, increasing $k_{\tilde{\sigma }}$ and applying the treatment also leads to less tumour growth and spread. Nonetheless, this still leads to a bigger tumour, and with much more spreading potential than in the mollifier case.

Furthermore, we performed experiments with different parameter values and found that the most compact and least invasive tumour spread was obtained when applying the chemoradiotherapy treatment to an initial maximum cancer cells density of 0.1, followed by applying the mollifier distribution to it within the oedema, with *k_R_* = 30. As shown in **Figure 8** top row this approach leads to barely any growth, and the tumour remains stable throughout the stages. Moreover, within the same scenario but considering the Gaussian distribution of cells within oedema with $k_{\tilde{\sigma }}$ =100, showcased in **Figure 8** bottom row, this also leads to less spread than in the previous experiment from **Figure 2**, but as showcased in the 3D panel of **Figure 8** (bottom row) for the Gaussian distribution simulation, the tumour is larger and spreads more than in the mollifier case from **Figure 8** (top row).

**Figure 9:**
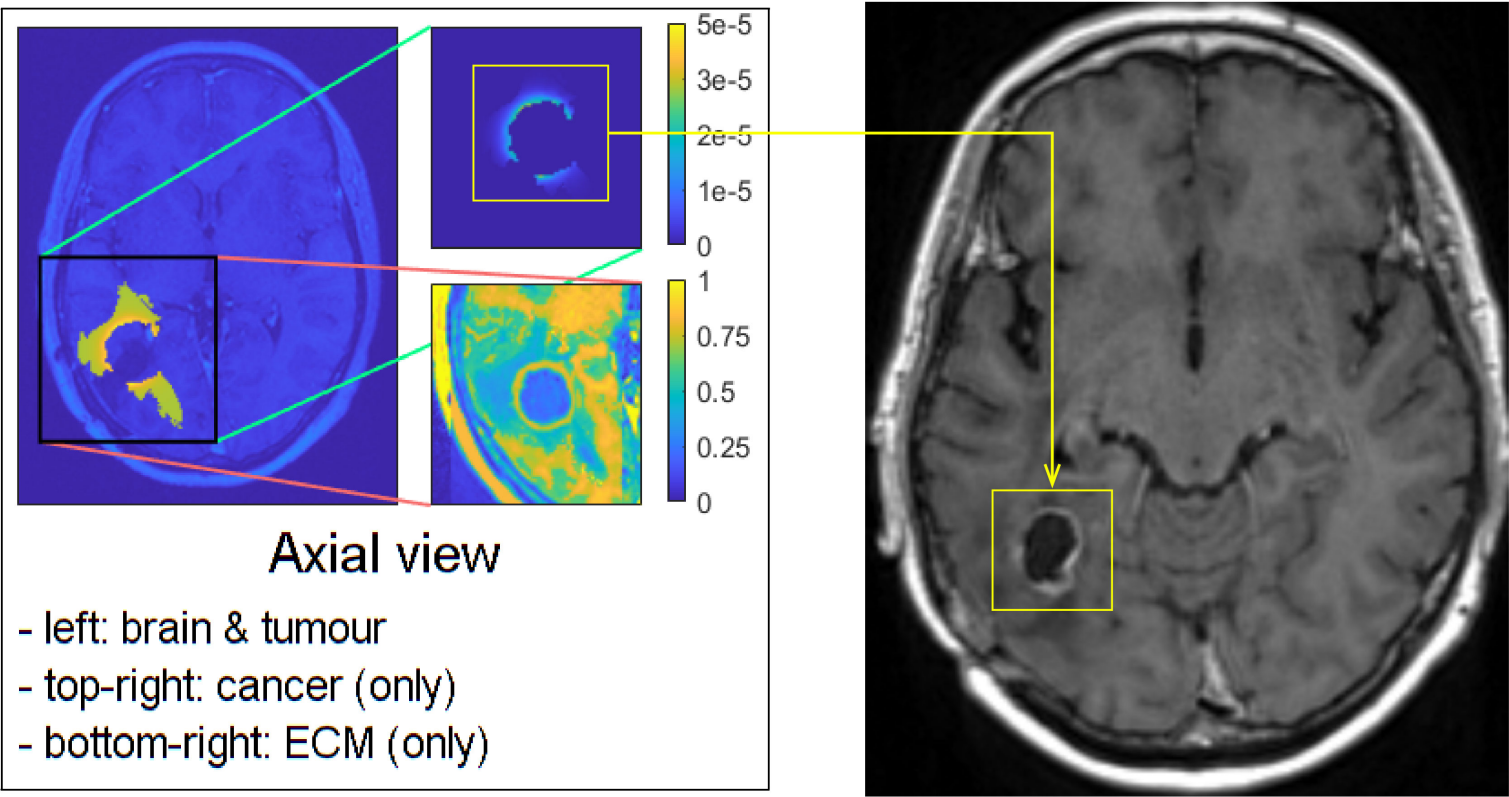
Visual comparison between one of our simulations and the MRI scan of the patient, taken 881 days after the first surgery.

Finally, during the course of various experiments, we observed an intriguing outcome. When applying the mollifier distribution to a specific set of values, the resulting outcome closely resembled the extent of the tumour from an MRI scan taken 881 days into the patient’s treatment, as evidenced by a visual comparison between the top–right image of our simulation and an actual MRI scan of the patient, as shown in **Figure 9**. This discovery guided us toward the subsequent phase of our goal: the comparative analysis of our simulations with MRI scans from this particular patient, enabled by the modification of $k_{R}$ or $k_{\tilde{\sigma }}$ according to the optimisation procedure from Section 2.2, so that our simulations can closely match the tumour extent observed in the given MRI scans from the considered patient.

### 3.4 Comparison between our simulations and the MRI data

A key objective of this work is to predict the growth dynamics of GBM tumours whilst incorporating a range of pre–operative and post–operative MRI scans from the specific patient into our analysis. This pursuit, crucial in the field of neuro–oncology, demands a thorough examination of the treatments received by these patients plus the analysis of the MRI scans. This examination is carried out through rigorous comparisons between our computational simulations and the existing MRI scans.

Starting from the baseline parameters set from **Table S1** in **Supplementary Material**, we use the optimisation procedure described in Section 2.2 to approximate the optimal *k_R_* or $k_{\tilde{\sigma }}$ (depending on the case considered), which ensure a good match between the MRI scans and the simulations. Then to properly compare our simulations to MRI scans, we start by aligning the simulation data with the corresponding MRI scan taken at a specific time in our timeline. Comparing the outlined tumour volume from the MRI, outlined under the supervision of KHI and MO, with our simulated cancer density, we calculate the absolute difference following the methods described in Section 2.2, such that Equation 19 is satisfied. If, at any time point, the cancer growth exceeds a set threshold and the disparities between the actual and predicted data are significant, the simulation is halted. Subsequently, *k_R_* or $k_{\tilde{\sigma }}$ are adjusted in a dyadic fashion until the simulation closely matches the real data, meeting our predefined threshold, which we set to be 5000 voxels, *i.e.*, if the difference between our simulation and the VOIs (from MRI scan data) is larger than 5000, then the simulation will stop.

This iterative refinement process ensures that our simulations accurately represent tumour dynamics observed in MRI scans, thereby advancing towards enhancing the reliability and applicability of our computational models.

#### 3.4.1 Utilising post–surgical MRI scans for more realistic tumour simulations

**Figure 10:**
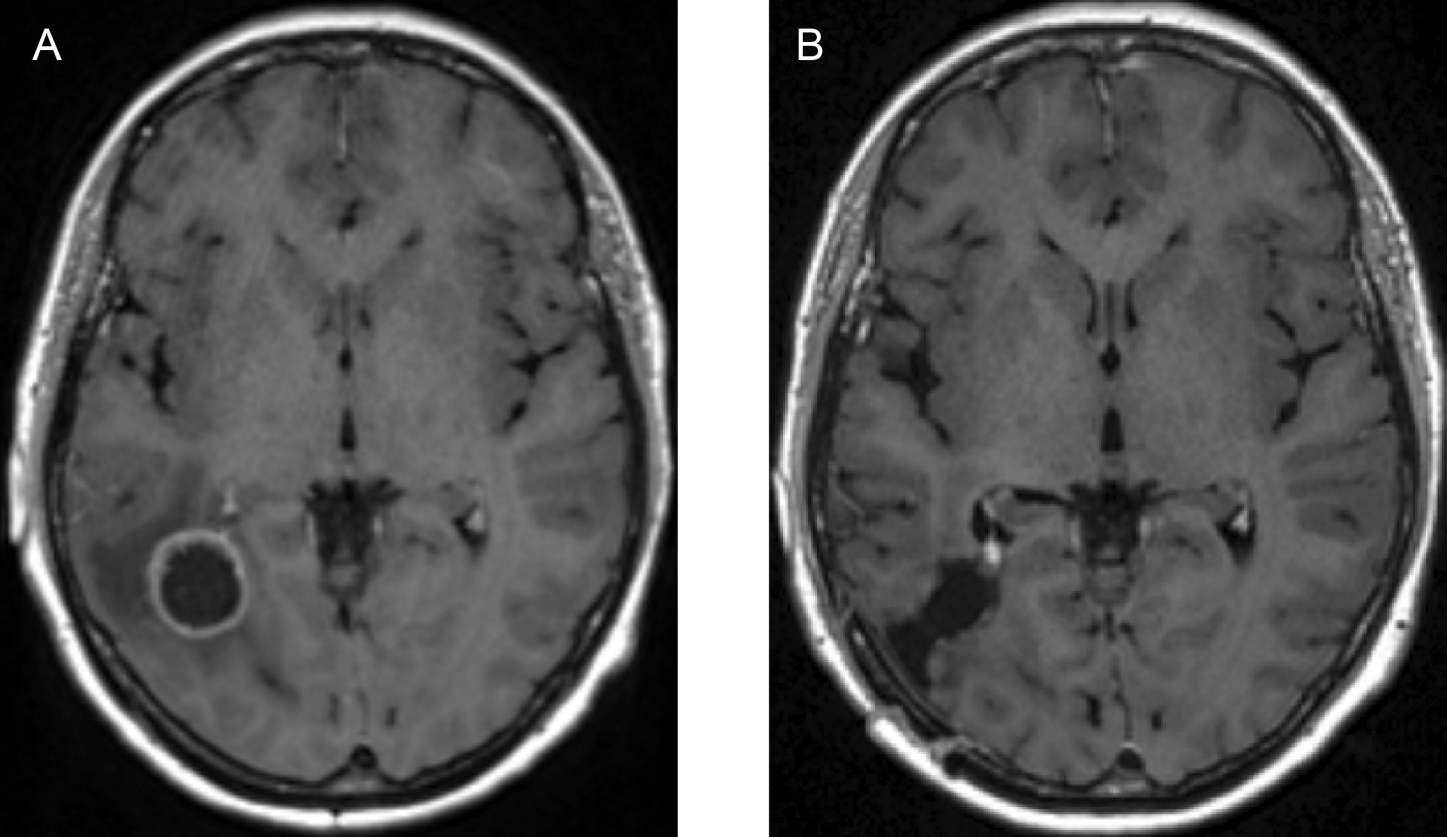
Image showing: **(A)** axial view of the T1+C pre–operative MRI scan, and **(B)** axial view of the post–operative MRI scan of the same patient, showcasing the anatomical differences of the brain structure due to the surgical intervention.

**Figure 11:**
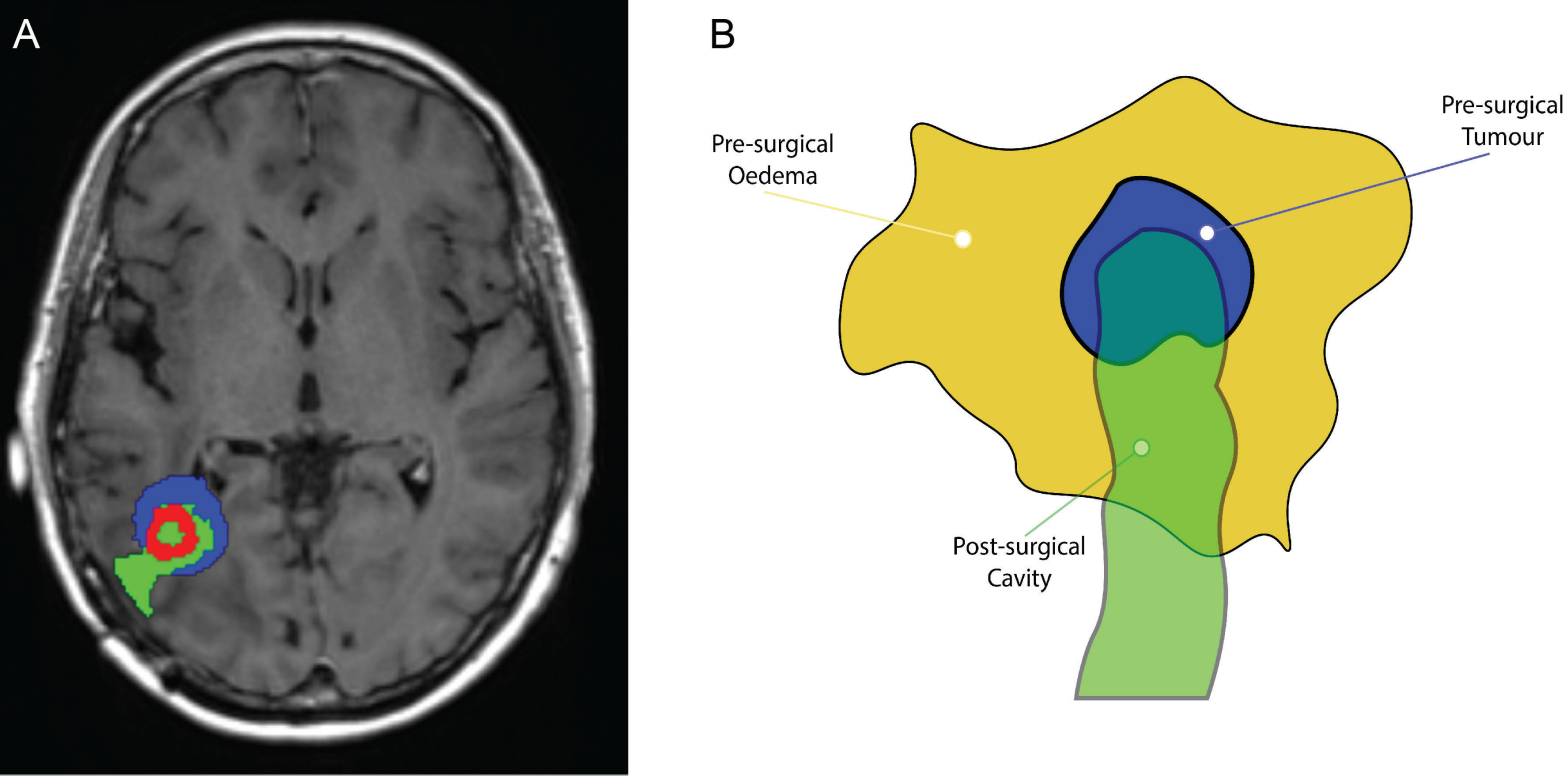
**(A)** Superimposition illustrating the spatial alignment of the pre–surgical original tumour (depicted in blue), the post–surgery surgical cavity (highlighted in green), and the recurrent tumour preceding the second surgical procedure (presented in red). **(B)** Schematic illustrating the dynamics of the three masks: the pre–surgical oedema mask (in dark yellow), the initial tumour mask (in blue), and the surgical cavity mask (in green). The brighter green region that is not overlapping with the oedema mask, is designated as zero, *i.e.*, no cancer will be located in this area.

In earlier stages of our research, we focused solely on the initial oedema volume before surgery and the main tumour size before any operation took place, as shown in **Figure 3**. However, while this approach was methodologically sound, it falls short when attempting to replicate the evolving changes observed in later MRI scans of the patient. These changes occur as the patient’s anatomy undergoes significant transformations due to surgery, as visually depicted in **Figure 10**. The patient in question experienced a noticeable reduction in the size of the original tumour site after surgical intervention. This reduction was followed by a recurrence of a smaller tumour, as shown in **Figure 11A**. Consequently, starting our simulations solely based on the initial tumour outline inevitably leads to a tumour size pattern that exceeds our expectations. To address this methodological challenge, we introduced a novel element to our initial conditions: the post–surgical cavity MRI scan VOI. This post–surgical MRI scan provides a clear view of the changes in brain anatomy following surgery, as depicted in **Figure 10**. By incorporating this post–operative anatomical data into our computational framework, we bridge the crucial gap between the pre–operative and immediate post–operative states. This enables a more precise and anatomically and physiologically realistic simulation of GBM tumour growth dynamics in the context of surgical interventions.

The mathematical implementation of this innovative volume addition requires a robust framework. We introduce three distinct masks: the pre–surgical oedema mask, the initial tumour mask, and the surgical cavity mask, as illustrated in **Figure 11B**. We introduce two new constants which indicate the presence or absence of cancer cells within these masks, represented as *α_it_* and *α_sc_*. Following the notation described in Section 2.2, we employ the mollifier distribution within the different masks to articulate this operation as follows:

- For the initial tumour mask: $c_{it}^{d_{x}}\left(\upsilon \right):=R{\left(n\left(x\right),k_{R}\right)}^{-1}\psi_{1}\left(\frac{\upsilon }{R\left(n\left(x\right),k_{R}\right)}\right)\alpha_{it},\;\upsilon \in \left[0,q\left(n\left(x\right),0\right)\right],$

- For the surgical cavity mask: $c_{sc}^{d_{x}}\left(\upsilon \right):=R{\left(n\left(x\right),k_{R}\right)}^{-1}\psi_{1}\left(\frac{\upsilon }{R\left(n\left(x\right),k_{R}\right)}\right)\alpha_{sc},\;\upsilon \in \left[0,q\left(n\left(x\right),0\right)\right].$

On the other hand, when using a Gaussian distribution, we have the following equations:

- For the initial tumour mask: $c_{it}^{d_{x}}\left(\upsilon \right)\propto N_{d_{x}}\left(0,\tilde{\sigma }\left(n\left(x\right),k_{\tilde{\sigma }}\right)\right)\alpha_{it},\;\upsilon \in \left[0,q\left(n\left(x\right),0\right)\right],$

- For the surgical cavity mask: $c_{sc}^{d_{x}}\left(\upsilon \right)\propto N_{d_{x}}\left(0,\tilde{\sigma }\left(n\left(x\right),k_{\tilde{\sigma }}\right)\right)\alpha_{sc},\;\upsilon \in \left[0,q\left(n\left(x\right),0\right)\right].$

As shown in the schematic diagram in **Figure 11B**, it is clear that the surgical cavity is slightly more elongated than the oedema mask. Therefore, regions where these volumes do not overlap are defined by setting their values to zero.

In essence, this mathematical framework equips our computational model with the ability to smoothly incorporate the complex interactions between the oedema, initial tumour, and surgical cavity VOIs. This leads to a more physiologically accurate simulation of GBM tumour growth dynamics, especially in the context of surgical procedures and chemoradiotherapy treatments.

**Figure 12:**
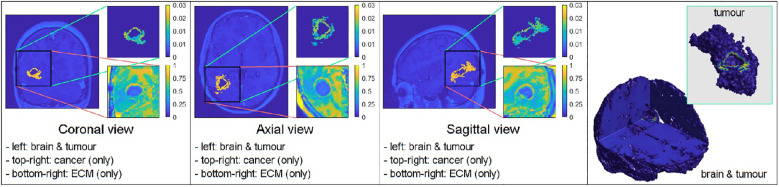
Simulation with the mollifier distribution using the three introduced masks (the pre–surgical tumour VOI, the pre–surgical oedema VOI, and the surgical cavity VOI), with *k_R_* = 20.

The outcome of the least square minimisation outlined in Section 2.2 led to the identification of the appropriate *k_R_*, that is then used in the mollifier–type distribution of cancer cells in the oedema for the considered patient. With the identified parameter *k_R_* = 20, the numerical simulation is shown in **Figure 12**.

**Figure 13:**
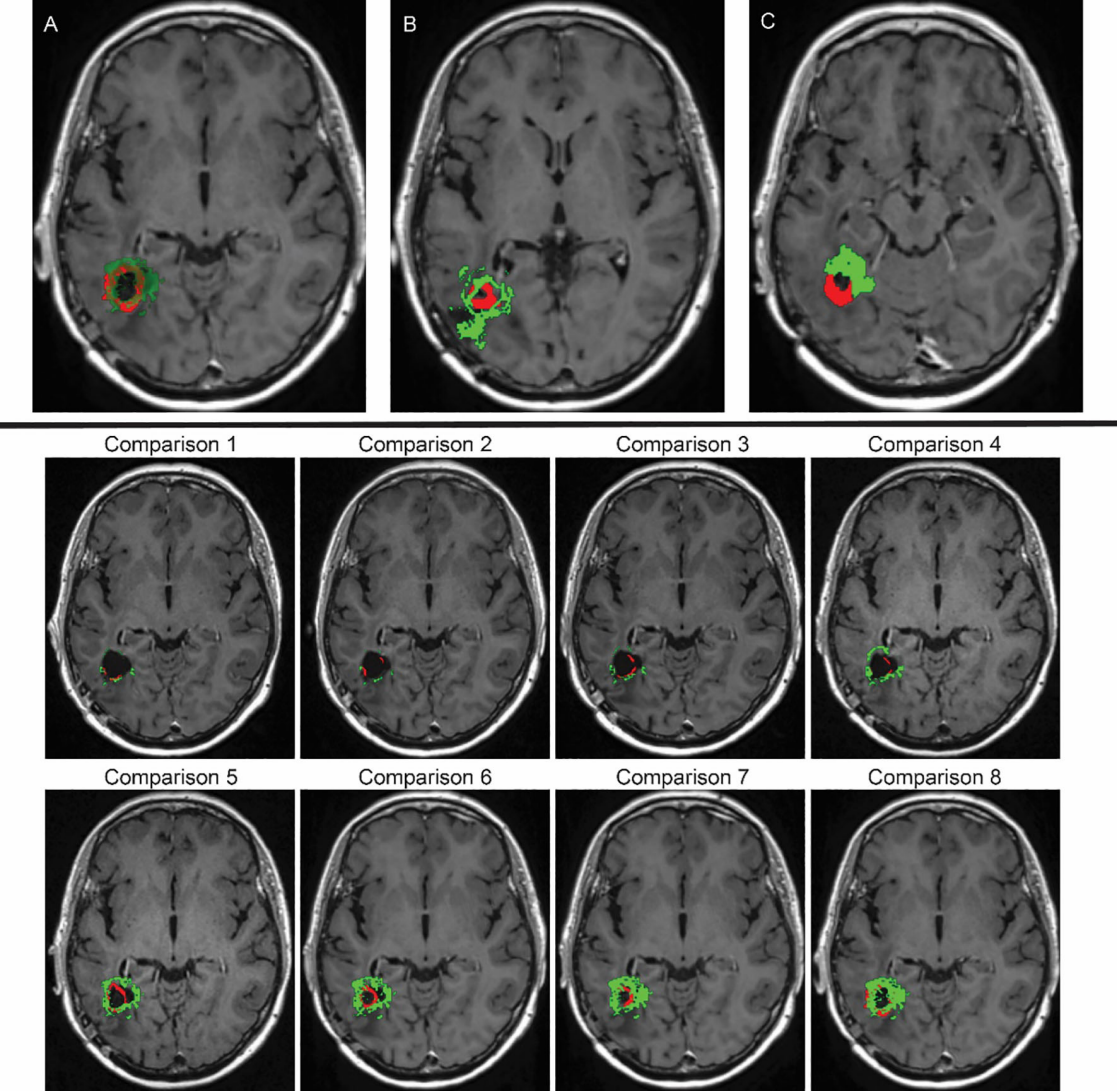
Top row: **(A)** Overlay depicting the spatial compatibility between our computational simulation (highlighted in green) and the MRI scan demonstrating GBM that recurred prior to the second surgery of the patient (emphasised in red), aligning with the data from the final MRI examination. **(B, C)** These overlays also involve our computational simulation (in green) and the recurred GBM pre–second–surgery MRI scan (in red). However, in this case, the alignment lacks a high degree of accuracy. Bottom two rows: Time evolution of the tumour recurrence (in red) and the simulation (in green), from month 6 (Comparison 1) into the treatment until month 26 (Comparison 8) from **Figure 5**. For each of these scans, a comparison was made between the real tumour growth and our simulations.

Notably, the tumour in these results is noticeably smaller and has a more compact spatial distribution. Importantly, this tumour is completely surrounded and confined within the boundaries outlined by the surgical cavity mask, which closely matches the patient’s MRI scans, as illustrated in **Figure 13A**, which represents an overlapping of our simulation (simulation of **Figure 12** at stage 44, in green) and the recurred GBM cancer (in red), overlaid over the corresponding MRI scan slice taken 881 days into the treatment (or over 28 months, as depicted in the bottom schematic of **Figure 5**). The simulation of our model closely aligns with real–world clinical observations for this particular MRI slice. This strong agreement demonstrates the model’s effectiveness and its potential for predicting relevant outcomes. While **Figure 13A** showcases a high degree of accuracy, it is important to acknowledge that not all MRI slices achieve this level of precision, as shown in **Figures 13B, C**.

This achievement marks a significant milestone in our effort to accurately replicate the complexities of GBM tumour growth in the presence of surgical interventions, treatment administrations, and cancer cell distributions within the oedema. This mathematical modelling contributes to our understanding of the clinical management of this very challenging medical condition.

## 4 Discussion

GBM, an extremely aggressive brain tumour with a low 5–year survival rate of only 7.2%, poses significant challenges in terms of treatment (1, 2). In the search for better therapies, mathematical modelling has emerged as a valuable approach. Despite established treatments like the Stupp protocol, GBM almost always recurs, driven by its invasive nature and peritumoural oedema infiltration, in some cases (9–12). Mathematical models provide a promising way to understand the complexities of GBM.

Our study investigates the connection between the swelling around the tumour (peritumoural oedema) and the distribution of GBM cells within the oedema, whilst using MRI data. Building upon the 3D multiscale moving–boundary framework we introduced earlier, we have incorporated the treatment history of a specific patient from Ninewells Hospital. By simulating how tumours typically grow, our research sets the stage for future experiments using MRI data and treatment histories collected from GBM patients. Ultimately, we aim to develop a mathematical model that incorporates the effects of chemoradiotherapy and investigates the distribution of GBM cells within the oedema with greater accuracy, whilst also taking into account the anatomical changes of the brain due to surgery.

In each simulation, we initiate the process by segmenting the oedema and pre–surgical tumour masks obtained from the MRI scans of the specific GBM patient. Crucially, we meticulously replicate and take account of the exact treatment protocol administered to this patient in our simulations, as showcased in **Figure 5**. Furthermore, we investigate two scenarios for how cancer cells are distributed within the oedema: the mollifier and Gaussian distributions. The resulting figures show various outcomes based on different values for *k_R_* and $k_{\tilde{\sigma }}$ . These simulations closely resemble what doctors see in real clinical cases, where recurrent GBM often has the highest concentration of cancer cells at the edge of the surgically removed area (41, 42, 48, 49). As illustrated in **Figures 1**, **2**, decreasing *k_R_* and $k_{\tilde{\sigma }}$ respectively, corresponds to increased tumour aggressiveness. Notably here, each of the two distributions considered for the cancer cell population within the oedema decays towards the outer boundary of the oedema - an aspect that is consistent with evidence derived from clinical patient histopathology samples (48–50). Moreover, the mollifier distribution does go to zero as we approach the outer oedema boundary, however, the Gaussian does not. This is due to the fact that oedema represents a topologically compact region (subset) in ℝ^3^ where only the mollifier can die out to zero, while the Gaussian distribution remains always strictly positive (above a minimal threshold level).

Our experiments, involving a range of parameter combinations and the application of the chemoradiotherapy treatment, have shown that the most controlled and least invasive tumour growth occurs when we start with a maximum cancer cell density of 0.1 and use the mollifier distribution, with *k_R_* = 30, to arrange the cancer cells within the oedema, as observed in **Figure 8**.

What is particularly noteworthy is that our simulations closely match MRI scans taken years into the treatment, as shown in **Figure 9**, suggesting good agreement between our model and real–world data. This promising finding motivated us to improve our methods to predict GBM growth dynamics by incorporating various pre–and post–operative MRI data along with treatment effects. Initially, we only focused on the initial pre–operative tumour and oedema regions. However, this approach fell short when trying to capture the dynamic changes that occur after surgery, as observed in **Figure 10**. To address this limitation, we introduced the most immediate and highest quality post–operative MRI data, such that the surgical cavity can be outlined without any complications, into our framework, bridging the gap between the pre–operative and post–operative states. This integration involved three masks (the pre–surgical oedema, the initial tumour, and a future representation of the surgical cavity), as shown in **Figure 11B**, regulated by constants and applying the mollifier or Gaussian distributions, according to the methods described in Section 2.2. This framework allowed for more precise simulations, as evidenced by the reduction in tumour size and spatial distribution, which closely matched the patient’s MRI scan taken 881 days after the first surgery, as seen in **Figure 13A**.

In conclusion, our model represents a significant advancement in our ability to predict how GBM tumours behave following surgery, treatment administration and the distributions of cancer cells within the oedema. By incorporating pre–operative and post–operative MRI scans and carefully considering patient treatment histories, we have developed a robust framework that accurately replicates the complex dynamics of GBM progression. This achievement not only enhances our understanding of this challenging disease but also opens up significant possibilities in the field of clinical management.

In terms of modelling challenges and limitations, our model has many unknown variables, which are either estimated from the relevant literature and/or are patient specific. Gathering more data from experimental studies that accurately measure the different parameters involved in GBM growth and invasion can help the model become more accurate. Furthermore, as evidenced in **Figure 13**, bottom images, the accuracy of the evolution of our simulations, which is being overlaid over the tumour recurrence over time, still needs to be further improved. While this work is meant to be a proof of concept, this model needs validation for its predictive potentials. This is something that we are currently working on, aiming to apply the model to other individuals from a larger patient cohort to explore its level of transferability.

This research reflects current advancements in GBM research by providing valuable insights into mathematical modelling and its potential to predict this aggressive disease. By translating these insights into improved treatments, we hope this work will lead to a significantly improved outlook for GBM patients.

## Data Availability

Permission for access to the data is available via request to the NHS Tayside Caldicott Guardian, and NHS Tayside Ethics and R&D Department. Requests to access the datasets should be directed to NHS Tayside Caldicott Guardian, and NHS Tayside Ethics and R&D Department.
