# Supplementary material for: Post–operative glioblastoma cancer cell distribution in the peritumoural oedema
Source: Front Oncol. 2024 Dec 12;14:1447010. doi: 10.3389/fonc.2024.1447010 (PMC11669604; doi:10.3389/fonc.2024.1447010)
Supplement: Supplementary file 1 [file Presentation1.pdf]

Supplementary Material

1 OUTSIDE TUMOUR BOUNDARY

Following the definition of $\partial\Omega_o(t)$ by Suveges et al. (2022), let $x \in \partial\Omega(t)$. Then, $x \in \partial\Omega_o(t)$ if and only if there exists $\phi_x : [0, \infty) \to \mathbb{R}^d$ such that the following properties hold true simultaneously:

$$
\begin{aligned}
&1) \quad \phi_x(0) = x, \\
&2) \quad \phi_x(s) \neq x, \forall s \in (0, \infty), \\
&3) \quad Im\phi_x \backslash \{x\} \subset \complement\Omega(t), \\
&4) \quad \lim_{s \to \infty} dist(\phi(s), \partial\Omega(t)) = \infty,
\end{aligned}
$$

where $\forall s \in (0, \infty)$, we have $dist(\phi(s), \partial\Omega(t)) := \inf_{x \in \partial\Omega(t)} \|\phi(s) - z\|_2$ and represents the Euclidean distance from $\phi(s)$ to $\partial\Omega(t)$.

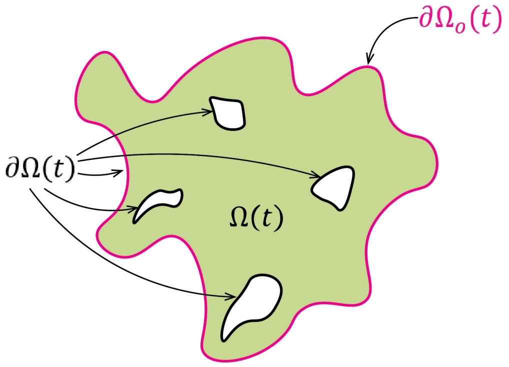

Figure S1: Schematic showing $\partial\Omega_o(t)$, the outer boundary highlighted with the dashed line.

2 TABLE OF PARAMETERS

Here we include the table with the baseline values for the parameters considered for our model at macro–scale. Furthermore, any other choices in the parameter values (*i.e.,* beyond the ones included in this table) is explained as appropriate in the text.

3 DEFINITION OF THE ANISOTROPIC DIFFUSION $\mathbb{D}_T(x)$ AND ADHESION $\mathcal{A}(x, t, \mathbf{u}, \theta_f)$ TERMS

The anisotropic diffusion term $\mathbb{D}_T(x)$ is defined as:

Parameters	Values	References
D_c	10^{-4}	(Suveges et al., 2022)
D_G	0.25	(Suveges et al., 2021)
D_σ	1	(Suveges et al., 2022)
D_m	2.5×10^{-3}	(Suveges et al., 2022)
$\mathcal{K}_{FA}$	100	(Suveges et al., 2021)
$\mathbf{S}_{\max}$	0.5	(Suveges et al., 2022)
$\mathbf{S}_{\min}$	0.01	(Suveges et al., 2022)
$\mathbf{S}_{cl}$	0.01	(Suveges et al., 2022)
$\mathbf{S}_{cF}$	0.3	(Suveges et al., 2022)
μ	0.25	(Suveges et al., 2022)
d	0.015	(Suveges et al., 2022)
d_σ	80	(Suveges et al., 2022)
β_l	3	(Suveges et al., 2022)
β_F	1.5	(Suveges et al., 2022)
σ_{nor}	0.4	(Suveges et al., 2022)
σ_p	0.35	(Suveges et al., 2022)
σ_n	0.2	(Suveges et al., 2022)
$\Psi_{p,\max}$	1	(Suveges et al., 2022)
$\Psi_{d,\max}$	5	(Suveges et al., 2022)
b	1.1	(Bashkirtseva et al., 2021)
s	2	(Bashkirtseva et al., 2021)
ζ	0.5	(Bashkirtseva et al., 2021)
W	130	Clinical estimate
α	0.06	(Qi et al., 2006; van Leeuwen et al., 2018)
β	0.006	(Qi et al., 2006; van Leeuwen et al., 2018)
β_{FChemo}	0.5	Estimated
β_{FRadio}	0.5	Estimated
β_{lChemo}	0.5	Estimated
β_{lRadio}	0.5	Estimated
R	0.15	(Suveges et al., 2022)
r	0.0016	(Suveges et al., 2022)
$f_{\max}$	0.636	(Suveges et al., 2022)
N_{radio}	30	Clinical estimate
N_{chemo}	132	Clinical estimate
$D(j_m)$, $\forall m = 1 \dots N_{radio}$	$2Gy$	Clinical estimate

Table S1. The baseline parameter values used for the numerical simulations.

$$\mathbb{D}_T(x) := D_c D_{WG}(x)\Big[\Big(r + (1-r)\left(\frac{\coth k(x)}{k(x)} - \frac{1}{k(x)^2}\right)\Big) I_3 + (1-r)\left(1 - \frac{3\coth k(x)}{k(x)} + \frac{3}{k(x)^2}\right)\phi_1(x)\phi_1^T(x)\Big], \quad \text{(S1)}$$

where, $D_c > 0$ is the diffusion coefficient, while $D_{WG}(\cdot)$ acts as a regulator term, addressing the well known fact that malignant glioma cells have higher motility in white matter than in grey matter (Chicoine and Silbergeld, 1995; Silbergeld and Chicoine, 1997; Swanson et al., 2000; Brooks et al., 2021), and is defined as:

$$D_{WG}(x) = \big((D_G g(x) + w(x)) * \psi_\rho\big)(x), \quad \text{(S2)}$$

The ratio between the motility regimes in grey and white matter is given here by $D_G \in [0,1]$, $g(x)$ and $w(x)$ are the grey and white matter densities, respectively, which are obtained from the T1 scan (Suveges et al., 2021). Further, $\psi_\rho(x) := \psi_3(x/\rho)/\rho^3$ is the mollifier induced by the standard mollifier ψ_3 defined in Appendix 5, and $*$ denotes the convolution operator. Furthermore, $r \in [0,1]$ is the extent of isotropic diffusion, I_3 is the 3×3 identity matrix. Moreover, $\lambda_1(x) \geq \cdots \geq \lambda_N(x)$ denote the eigenvalues, while $\phi_1(x), \cdots, \phi_N(x)$ represent the corresponding eigenvectors. Finally, $k(x)$ is given by

$$k(x) := \mathcal{K}_{FA} FA(x),$$

with $\mathcal{K}_{FA} \geq 0$ measuring the sensitivity of the cells to the direction of the environment, while $FA(x)$ stands for the *fractional anisotropy index* (Engwer et al., 2014; Suveges et al., 2021) and is defined as

$$FA(x) := \sqrt{\frac{(\lambda_1(x) - \lambda_2(x))^2 + (\lambda_2(x) - \lambda_3(x))^2 + (\lambda_1(x) - \lambda_3(x))^2}{2(\lambda_1^2(x) + \lambda_2^2(x) + \lambda_3^2(x))}}.$$

The second term in Equation (7), namely $\nabla[c\mathcal{A}(x,t,\mathbf{u},\theta_f)]$, describes adhesion processes that bias the movement of the cell population due to the adhesion bonds that the migratory cells establish with both the surrounding cell and the ECM components. Introduced in Shuttleworth and Trucu (2019) and expanded later in Suveges et al. (2021), the non–local flux term considers the interactions of cancer cells within a sensing region $\mathbf{B}(0,R)$, with radius $R > 0$, described by:

$$\mathcal{A}(x,t,\mathbf{u},\theta_f) := \frac{1}{R}\int_{\mathbf{B}(0,R)} \mathcal{K}(y)\Big[n(y)(\mathbf{S}_{cc}c(x+y,t) + \mathbf{S}_{cl}l(x+y,t)) + \hat{n}(y,\theta_f(x+y,t))\mathbf{S}_{cF}(x+y,t)\Big][1-\rho(\mathbf{u})]^+ dy, \quad \text{(S3)}$$

where $\mathbf{S}_{cc}, \mathbf{S}_{cl}, \mathbf{S}_{cF} > 0$ are the cell–cell, cell–non–fibrous ECM and cell–fibrous ECM adhesion strength coefficients, respectively. $\mathbf{S}_{cc}$ is positively correlated to the levels of extracellular Ca^{2+} ions. Hence, we describe the cell–cell bonds as:

$$\mathbf{S}_{cc} := \mathbf{S}_{\min} + (\mathbf{S}_{\max} - \mathbf{S}_{\min})\exp\Big[1 - \frac{1}{1-(1-l(x,t))^2}\Big],$$

with $\mathbf{S}_{\min} > 0$ and $\mathbf{S}_{\max} > 0$ are the minimum and maximum levels of Ca^{2+} ions (Suveges et al., 2021, 2022). Furthermore, the gradual weakening of these bonds are represented by using a radially symmetric kernel $\mathcal{K}(\cdot)$ given by:

$$\mathcal{K}(y) = \psi_1\Big(\frac{y}{R}\Big), \qquad \forall y \in \mathbf{B}(0,R),$$

where $\psi_1(\cdot)$ is the standard mollifier defined in Appendix 5. Moreover, in Equation (S3), $n(\cdot)$ and $\hat{n}(\cdot,\cdot)$ are the unit radial vector and unit radial vector biased by the oriented ECM fibres (Suveges et al., 2021), described mathematically as

$$n(y) := \begin{cases} \frac{y}{\|y\|_2} & \text{if } y \in \mathbf{B}(0,R)\setminus\{0\}, \\ 0 & \text{if } y = 0, \end{cases}$$

$$\hat{n}(y,\theta_f(x+y,t)) := \begin{cases} \frac{y+\theta_f(x+y,t)}{\|y+\theta_f(x+y,t)\|_2} & \text{if } y \in \mathbf{B}(0,R)\setminus\{0\}, \\ 0 & \text{if } y = 0. \end{cases}$$

Finally, to prevent overcrowded regions contributing to cell migration, we have a limiting term $[1-\rho(\mathbf{u})]^+ := \max(0, 1-\rho(\mathbf{u}))$ (Suveges et al., 2021).

4 DEFINITION AND DETAILS OF THE ECM FIBRES VECTOR FIELD INDUCED FROM MICRO–FIBRES

The fibre vector field $\theta_f(x,t)$ is defined as:

$$\theta_f(x,t) := \frac{1}{\lambda(\delta Y(x))} \int\limits_{\delta Y(x)} f(z,t)dz \cdot \frac{\theta_{f,\delta Y(x)}(x,t)}{\|\theta_{f,\delta Y(x)}(x,t)\|_2}. \tag{S4}$$

Here, $f(z,t)$ is the micro–scale mass density of micro–fibres distributed on a micro–domain $\delta Y(x) := x + \delta Y$ of appropriate micro–scale size $\delta > 0$, while $\lambda(\cdot)$ is the usual Lebesque measure in $\mathbb{R}^3$. Further, $\theta_{f,\delta Y(x)}(\cdot,\cdot)$ is the revolving barycentral orientation given by:

$$\theta_{f,\delta Y(x)}(x,t) := \frac{\int\limits_{\delta Y(x)} f(z,t)(z-x)dz}{\int\limits_{\delta Y(x)} f(z,t)dz}.$$

5 THE STANDARD MOLLIFIER AND THE PER-DAY RADIO AND CHEMO SCHEDULING

The form of the standard symmetric mollifier on $\psi_n : \mathbb{R}^n \to \mathbb{R}_+$, $n \in \{1,3\}$, used in this manuscript is given by:

$$\psi_n(x) = \begin{cases} \exp\left(\frac{-1}{1-\|\mathbf{x}\|_2^2}\right) & , \quad x \in \mathbf{B}(0,1) \\ 0 & , \quad x \notin \mathbf{B}(0,1), \end{cases} \tag{S5}$$

Finally, the overlapping effect for both chemo- and radio- therapy delivery, is described by

$$\begin{aligned} \psi_{i_k}^{chemo}(t) &= \psi^{scheduling}(i_k, t), \qquad \forall k \in \{1 \ldots N_{chemo}\}, \\ \psi_{j_m}^{radio}(t) &= \psi^{scheduling}(j_m, t), \qquad \forall m \in \{1 \ldots N_{radio}\}, \end{aligned} \tag{S6}$$

with

$$\psi^{scheduling}(p, t) := \begin{cases} e^{\left(\frac{1}{d^2} - \frac{1}{d^2 - (t - T_p)^2}\right)} & \text{if } t \in (T_p, T_p + d), \\ e^{\left(\frac{1}{l^2} - \frac{1}{l^2 - (t - T_p)^2}\right)} & \text{if } t \in (T_p - l, T_p), \\ 0 & \text{if } t \in (-\infty, T_p - l) \cup (T_p + d, +\infty), \end{cases}$$

REFERENCES

Bashkirtseva, I., Ryashko, L., López, Á. G., Seoane, J. M., and Sanjuán, M. A. F. (2021). The effect of time ordering and concurrency in a mathematical model of chemoradiotherapy. *Communications in Nonlinear Science and Numerical Simulation* 96, 105693. doi:10.1016/j.cnsns.2021.105693

Brooks, L. J., Clements, M. P., Burden, J. J., Kocher, D., Richards, L., Devesa, S. C., et al. (2021). The white matter is a pro-differentiative niche for glioblastoma. *Nature Communications* 12, 2184. doi:10.1038/s41467-021-22225-w

Chicoine, M. R. and Silbergeld, D. L. (1995). Assessment of brain tumor cell motility in vivo and in vitro. *Journal of Neurosurgery* 82, 615–622. doi:10.3171/jns.1995.82.4.0615

Engwer, C., Hillen, T., Knappitsch, M., and Surulescu, C. (2014). Glioma follow white matter tracts: a multiscale dti-based model. *Journal of Mathematical Biology* 71, 551–582. doi:10.1007/s00285-014-0822-7

Qi, X. S., Schultz, C. J., and Li, X. A. (2006). An estimation of radiobiologic parameters from clinical outcomes for radiation treatment planning of brain tumor. *International Journal of Radiation Oncology*Biology*Physics* 64, 1570–1580. doi:10.1016/j.ijrobp.2005.12.022

Shuttleworth, R. and Trucu, D. (2019). Multiscale modelling of fibres dynamics and cell adhesion within moving boundary cancer invasion. *Bulletin of Mathematical Biology* 81, 2176–2219. doi:10.1007/s11538-019-00598-w

Silbergeld, D. L. and Chicoine, M. R. (1997). Isolation and characterization of human malignant glioma cells from histologically normal brain. *Journal of Neurosurgery* 86, 525–531. doi:10.3171/jns.1997.86.3.0525

Suveges, S., Eftimie, R., and Trucu, D. (2022). Re-polarisation of macrophages within collective tumour cell migration: A multiscale moving boundary approach. *Frontiers in Applied Mathematics and Statistics* 7. doi:10.3389/fams.2021.799650

Suveges, S., Hossain-Ibrahim, K., Steele, J. D., Eftimie, R., and Trucu, D. (2021). Mathematical modelling of glioblastomas invasion within the brain: A 3d multi-scale moving-boundary approach. *Mathematics* 9, 2214. doi:10.3390/math9182214

Swanson, K. R., Alvord, E. C., and Murray, J. D. (2000). A quantitative model for differential motility of gliomas in grey and white matter. *Cell Proliferation* 33, 317–329. doi:10.1046/j.1365-2184.2000.00177.x

van Leeuwen, C. M., Oei, A. L., and Crezee, J. e. a. (2018). The alfa and beta of tumours: a review of parameters of the linear-quadratic model, derived from clinical radiotherapy studies. *Radiation Oncology* 13, 96. doi:10.1186/s13014-018-1040-z
